# Synthesis of aromatic lactone analogues of Lipoxin A4

**DOI:** 10.1186/s13104-022-05917-4

**Published:** 2022-02-09

**Authors:** Aya Ismael, Muhammad Zeeshan, Jørn H. Hansen

**Affiliations:** Department of Chemistry, Chemical Synthesis and Analysis Division, UiT The Arctic University of Norway, 9037 Tromsø, Norway

**Keywords:** Lipoxin analogues, Aromatics, Wittig olefination, Lactones

## Abstract

**Objective:**

Synthesis of novel aromatic Lipoxin A4 lactone analogues.

**Results:**

Novel *para-*substituted aromatic lactone analogues of Lipoxin A4 have been synthesized in a convergent manner with six steps in the longest linear sequence in 12–13% yields, employing 2-deoxy-d-ribose as a chiral pool starting material and the classical *E-*selective Wittig olefination.

**Supplementary Information:**

The online version contains supplementary material available at 10.1186/s13104-022-05917-4.

## Introduction

Lipoxins (LXs) are naturally occurring oxygenated derivatives of arachidonic acid that are produced and act at sites of inflammation [1–6]. Lipoxin A4 (LXA4) and Lipoxin B4 (LXB4) were first reported by Serhan and Samuelsson in 1984 [7]. They are biosynthesized from arachidonic acid via lipoxygenases and both LXA4 and LXB4 are known to exhibit potent and selective anti-inflammatory activity. Even though LXs play a vital role in the resolution of inflammation, their use in therapeutics is compromised because of their rapid metabolism [8–11]. This instability dramatically reduces the potential of LXs to be used as pharmacological agents, thus providing the impetus for us and others to develop syntheses of more stable analogues.

In recent decades, significant progress has been made in the synthesis of lipoxin-analogues. In the first reported synthesis by Petasis et al., the triene core of LXA4 was replaced by a more stable benzene ring, which introduced metabolic stability while conserving crucial biological activity [12, 13]. A stereoselective synthesis of aromatic LXA4 and LXB4 was reported by O’Sullivan et al. where they utilized a series of advanced reactions (Sharpless epoxidation, Pd-mediated Heck coupling and diastereoselective reductions) to achieve the desired stereochemistry. The resulting analogs exhibited increased phagocytotic activity compared to the native LXA4 [6]. A number of other synthetic strategies have also been reported [14–21]. In this paper, we describe a different and potentially more flexible approach to the synthesis of aromatic lipoxin analogues in their lactone forms using simple and available starting materials and the classical Wittig olefination to craft the central double bond. This research note reports on the performance of our synthetic approach to two novel lipoxin A4 analogues.

## Main text

### Results and discussion

**Fig. 1:**
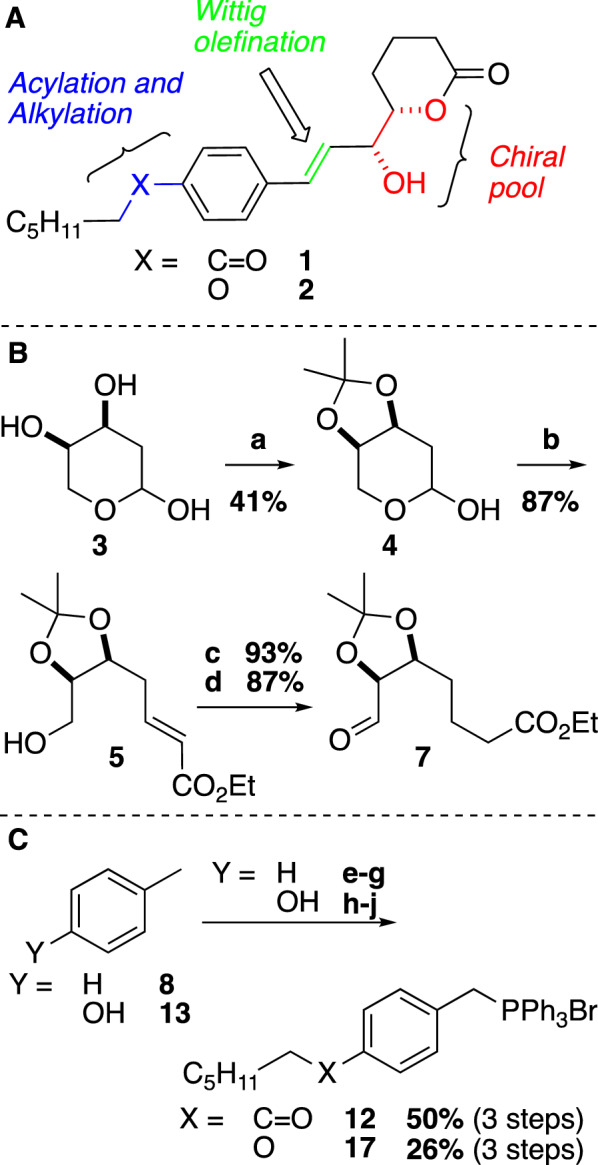
**A** Key synthetic strategies towards the target lactones. **B** Synthesis of key intermediate **7**; a) 2,2-dimethoxypropane, DMF, MS 5 Å, 0 °C, 3 h, then rt overnight, 41%; b) Ph_3_PCHCO_2_Et, benzoic acid, toluene, reflux, 87%; c) H_2_ (1 atm), Pd/C, EtOAc, rt, 93%; d) SO_3_-Pyr, Et_3_N/DMSO, DCM, rt, 87%. **C** Synthesis of key phosphonium salts **12** and **17**; e) Heptanoyl chloride, AlCl_3_, DCM, 0 °C, r.t, 30 min – 1 h, 73%; f) NBS, THF, H_2_O, hv, r.t, 83%; g) PPh_3_, acetone, reflux, 3 h, 82%. h) Heptyl bromide, K_2_CO_3_, CH_3_CN, 24 h, reflux, 72%, or; KOH, EtOH, H_2_O, 24 h, reflux, 76%; i) NBS, Bz_2_O_2_, DCM, reflux, 9 h, 48%. j) = g), 72%

We envisioned a synthetic strategy as outlined in Fig. 1A in which the aliphatic side chain would be connected via Friedel-Craft acylation and phenolate alkylation to generate the ketone and ether analogues **1** and **2**, respectively. The chiral diol moiety can be harvested from the chiral pool via an enantiomerically pure, commercially available 2-deoxy-D-ribose. The central double bond could be crafted via an *E*-selective Wittig-olefination.

The chiral pool fragment is introduced from aldehyde **7** (Fig. 1B), set up for the key Wittig olefination, which was synthesized starting from commercial 2-deoxy-D-ribose (**3**). In a four-step procedure, protecting the diol moiety as a cyclic acetal via acid-catalysis, followed by a tandem ring-opening/Wittig olefination, Pd-catalyzed hydrogenation of the α, β-unsaturated double bond and a Parikh-Doering oxidation of the resulting alcohol, the aldehyde **7** was formed in overall 29% yield from **3**. The appropriate phosphonium salts for the key Wittig olefination (**12** and **17**) were generated from toluene (**8**) and *p*-cresol (**13**), respectively (Fig. 1C). **8** was subjected to Friedel-Craft acylation with heptanoyl chloride, followed by light-induced benzylic radical bromination and subsequent nucleophilic displacement with triphenylphosphine to generate ketone **12** in 50% yield over 3 steps. **13** was treated with hexyl bromide and base, followed by benzylic bromination with *N*-bromosuccinimide (NBS) and the final nucleophilic displacement with triphenylphosphine to form phosphonium salt **17** in overall 26% over 3 steps. The typical Wohl-Ziegler benzylic mono-bromination could not be achieved in the system with the electron-donating alkoxy-group. An acceptable conversion to the benzylic bromide was achieved with NBS and a catalytic amount of dibenzoyl peroxide in the presence of dichloromethane (48% yield). A number of reported conditions for phosphonium salt formation initially failed for the formation of **17** but, it was found that a relatively short reaction time (3 h in refluxing acetone) was crucial to achieve 72% yield in this step.

**Fig. 2:**
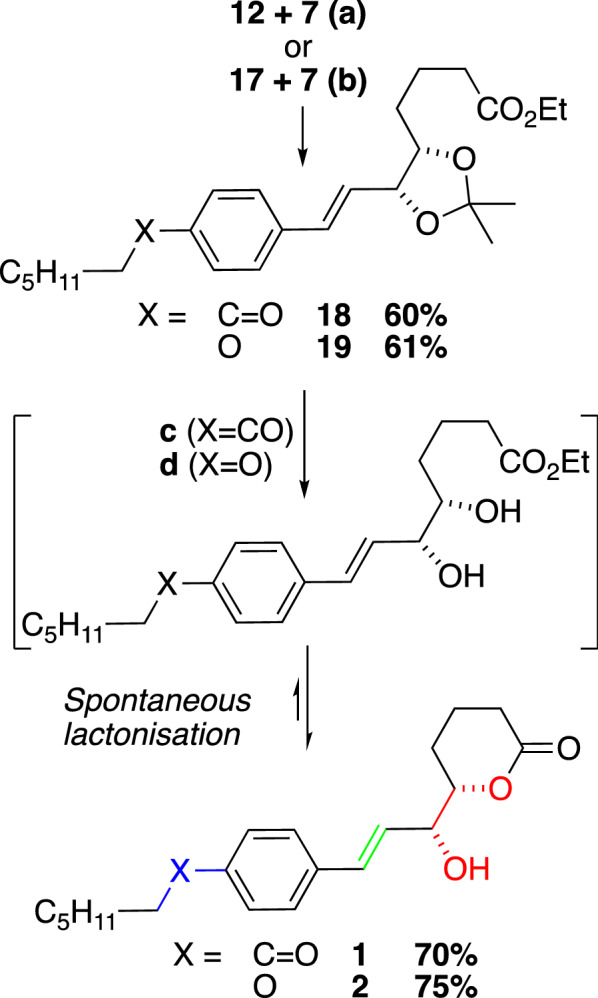
**a** K_2_CO_3_, THF, 0 °C, 1h – r.t. , 60%; **b** K_2_CO_3_, THF, 0 °C, 1h – r.t, 61%; **c** 1N HCl, THF, 0 °C - r.t. , 70%. **d** 1N HCl, THF, 0 °C - r.t, 75%

The phosphonium salts **12** and **17** were treated with base and the resulting ylenes coupled with aldehyde **7** in Wittig olefinations to yield the desired products **18** and **19** in 60% and 61% yields respectively (Fig. 2). We were unable to extract the *E/*Z-ratios from the Wittig olefination steps from the raw data but minor peaks appear in spectra of purified materials. The final removal of the acetal group from both **18** and **19** under acidic conditions gave 70% and 75% yields of the target lactones **1** and **2,** respectively. The unprotected diol esters were not detected directly but are likely intermediates in the formation of the lactones via spontaneous cyclization.

In summary, we have successfully synthesized two novel *trans-*substituted aromatic lactone analogues of Lipoxin A4 in a convergent manner with six steps in the longest linear sequence using simple transformations and affordable reagents in overall 12–13% yield.

### Methods

All reagents and solvents were purchased from commercial sources. The progress of the reactions were monitored by thin-layer chromatography (TLC) using Merck pre-coated silica gel plates (60 F_254_). The TLC plates were visualized using either ultraviolet light or by immersion in a solution of phosphomolybdic acid, followed by heating. For preparative purification, flash column chromatography was carried out using silica gel from Merck (Silica gel 60, 0.040–0.063 mm). The intermediates and products were characterized by NMR on a 400 MHz Bruker Avance III HD. Chemical shifts (*δ*) are reported in ppm relative to the residual solvent peak (CDCl_3_: *δ*_H_ 7.26 and *δ*_C_ 77.16; methanol-d_4_: *δ*_H_ 3.31 and *δ*_C_ 49.00, deuterium oxide: δ_H_ 4.79 and *δ*_C_ 49.00; DMSO-*d*_6_ *δ*_H_ 2.51 and *δ*_C_ 39.52). The raw data was analysed with MestReNova (Version 10.0.2–15,465). High-resolution mass spectra were obtained on a Thermo electron LTQ Orbitrap XL spectrometer, which was operated in electrospray ionization mass spectrometry (ESI) mode. The data was analyzed with Thermo Scientific Xcalibur software. Infrared spectra were recorded on a Varian 700e FT-IR spectrometer and bands are reported in wavenumbers (cm^−1^).

Detailed synthetic procedures and analytical data can be found in Additional file 1.

## Limitations

The initial protection of the chiral pool sugar **3** is difficult to achieve in high yields and is a weak point in this synthetic strategy. Moreover, the benzylic radical bromination of electron-rich aromatics is a clear limitation of the synthesis of the phosphonium salts. Alternative strategies for this could be considered, e.g. direct chloromethylation. A third limitation appears to be that isomers generated in the key Wittig olefination step are difficult to separate.

## Supplementary Information

**Additional file 1.** Experimental procedures, equipment, spectroscopic and analytical data for compounds presented in the manuscript.

## Data Availability

Spectroscopic data for all intermediates and final products can be found in a separate additional file and is also available from the corresponding author upon request.

## Abbreviations

LXs: Lipoxins
LXA4: Lipoxin A4
LXB4: Lipoxin B4
DMF: Dimethyl formamide
MS: Molecular sieves
rt: Room temperature
DMSO: Dimethyl sulfoxide
DCM: Dichloromethane
NBS: N-Bromosuccinimide
THF: Tetrahydrofuran
Bz: Benzoyl
TLC: Thin-Layer Chromatography
NMR: Nuclear Magnetic Resonance
FT-IR: Fourier Transform-Infrared Spectroscopy
