# Supplementary material for: Synthesis of aromatic lactone analogues of Lipoxin A4
Source: BMC Res Notes. 2022 Feb 9;15:30. doi: 10.1186/s13104-022-05917-4 (PMC8827294; doi:10.1186/s13104-022-05917-4)
Supplement: Supplementary file 1 — Additional file 1. Experimental procedures, equipment, spectroscopic and analytical data for compounds presented in the manuscript. [file 13104_2022_5917_MOESM1_ESM.pdf]

## Experimental Section

All reagents and solvents were purchased from commercial sources. The progress of the reactions were monitored by thin-layer chromatography (TLC) using Merck pre-coated silica gel plates (60 $F_{254}$). The TLC plates were visualized using either ultraviolet light or by immersion in a solution of phosphomolybdic acid, followed by heating. For preparative purification, flash column chromatography was carried out using silica gel from Merck (Silica gel 60, 0.040 - 0.063 mm). The intermediates and products were characterized by NMR on a 400 MHz Bruker Avance III HD. Chemical shifts ($\delta$) are reported in ppm relative to the residual solvent peak ($CDCl_3$ : $\delta_H$ 7.26 and $\delta_C$ 77.16; methanol-$d_4$ : $\delta_H$ 3.31 and $\delta_C$ 49.00, deuterium oxide: $\delta_H$ 4.79 and $\delta_C$ 49.00; DMSO-$d_6$ $\delta_H$ 2.51 and $\delta_C$ 39.52). The raw data was analysed with MestReNova (Version 10.0.2-15465). High-resolution mass spectra were obtained on a Thermo electron LTQ Orbitrap XL spectrometer, which was operated in electrospray ionization mass spectrometry (ESI) mode. The data was analyzed with Thermo Scientific Xcalibur software. Infrared spectra were recorded on a Varian 700e FT-IR spectrometer and bands are reported in wavenumbers ($cm^{-1}$).

### (3a*R*,7a*S*)-2,2-dimethyltetrahydro-4*H*-[1,3]dioxolo[4,5-*c*]pyran-6-ol (4)

2-deoxy-D-ribose 3 (3 g, 22.36 mmol) was dissolved in DMF (10 mL) in a dry and clean round bottomed flask equipped with molecular sieves and stirring bar. The mixture stirred under $N_2$ gas at 0°C before 2-Methoxypropene (3.225 g, 44.72 mmol) was slowly added to it. In another flask, pyridinium *p*-toluenesulfonate (0.50 g, 20.12 mmol) was dissolved in minimum amount of DMF and left stirring for 5 min before addition to the reaction mixture at 0°C. The reaction continued stirring at 0°C for 3h, then it was left overnight at room temperature. The product was filtered by suction, washed several times with DCM and then concentrated *in vacuo*. The crude was purified by column chromatography (pentane/ethyl acetate, 1:1) affording a colourless oil (1.751 g, 41 %) of the protected sugar 4. TLC; $R_f$ = 0.20 (heptane/ethyl acetate, 1:1); HRMS (ESI) m/z: $[M+Na]^+$ Calcd. for $C_8H_{14}O_4Na$ 197.0800, found 197.0800 and $[M+MeOH]^+$ 299.1000; $^1$H-NMR (DMSO-d6, 400 MHz) δ =6.27 (d, *J*=8 Hz, 1H), 4.97-4.93 (m,1H), 4.38-4.34 (m, 1H), 4.08-4.03 (m, 1H), 3.80 (dd, *J*= 4, 16Hz, 1H), 3.47 (dd, *J*= 4, 16Hz, 1H), 1.97-1.92 (m, 1H), 1.69-1.62 (m, 1H), 1.38 (s, 3H), 1.25 (s, 3H) ;$^{13}$C-NMR (DMSO-d6, 100 MHz) δ 107.6, 90.0, 70.9, 70.3, 61.2, 32.8, 27.4, 25.5. The data is consistent with the literature .[14, 28]

**Ethyl (*E*)-4-((4*S*,5*R*)-5-(hydroxymethyl)-2,2-dimethyl-1,3-dioxolan-4-yl) but-2-enoate (5)**

The protected sugar 4 (0.787 g, 4.51 mmol) was dissolved in toluene (10 mL) and refluxed for 10 minutes before the benzoic acid (0.0276 g, 0.226 mmol) was added to the reaction mixture. A solution of (Carboxymethylene)triphenylphosphorane (2.361 g, 6.78 mmol) in toluene was then added to the reaction mixture and the reaction was left refluxing at 90 °C for 2h. The reaction was monitored by TLC. Upon completion, the solvent was evaporated on a rotavapor. The crude was washed with diethyl ether to remove the excess of the Wittig salt. The resulted oil was purified by column chromatography (pentane/ ethyl acetate, 1:1) affording UV active colourless oil (0.96 g, 87%) of the alkene 5.TLC; $R_f$= 0.30; HRMS (ESI) m/z: $[M+Na]^+$ Calcd. for $C_{12}H_{20}O_5Na$ 267.1200 found 267.1200; $^1$H-NMR ($CDCl_3$, 400 MHz) δ =6.97-6.90 (m, 1H), 5.89 (d, *J*=16 Hz, 1H), 4.29-4.24 (m, 1H), 4.20-4.13 (m, 3H), 3.63 (d, *J*=8 Hz, 2H), 2.53-2.39 (m, 2H), 2.23 (d, *J*=8, 1H), 1.44 (s, 3H), 1.33 (s, 3H), 1.25 (t, *J*=8 Hz, 3H) ;$^{13}$C-NMR ($CDCl_3$, 100 MHz) δ=166.3, 144.6, 123.7, 108.6, 77.6, 75.5, 61.5, 60.4, 32.5, 28.1, 25.4, 14.3. The data is consistent with the literature .[(14)]

**Ethyl 4-((4*S*,5*R*)-5-(hydroxymethyl)-2,2-dimethyl-1,3-dioxolan-4-yl) butanoate (6)**

The alkene 5 (0.568 g, 2.32 mmol) was dissolved in ethyl acetate (10 mL) in a 25 mL round bottomed flask. The mixture was degassed by vacuum/argon cycles (5 times) before addition of Pd/C (10%). The reaction mixture was then evacuated and back filled with $H_2$ (2 times) before it was left stirring at room temperature under $H_2$ gas (1mp) for 5h. The reaction progress was monitored by TLC. Upon completion the reaction mixture was filtered through a Celite bed to capture the catalyst. The filtrate was collected and the solvent was evaporated in a rotavap affording (0.55 g, 93%) UV inactive colourless oil of 6. TLC; $R_f$ = 0.3 (heptane/ethylacetate, 1:1); HRMS (ESI) m/z: $[M+Na]^+$ Calcd. For $C_{12}H_{22}O_5Na$ 269.1400 , found 269.1350; $^1$H-NMR ($CDCl_3$, 400 MHz) δ = 4.13-4.08 (m, 5H), 3.59 ( d, *J* = 8 Hz, 2H), 2.34 (q, *J*= 4Hz, 2H), 2.14 (d, *J*= 8 Hz, 1H), 2.02 (s, 1H), 1.84-1.80 (m, 1H), 1.69-1.65 (m, 1H), 1.59-1.55 (m, 2H), 1.44 (s, 3H), 1.34 (s, 1H), 1.25 (t, *J*= 8Hz, 4H);$^{13}$C-NMR ($CDCl_3$, 100 MHz) δ=173.2, 107.9, 77.7, 61.5, 60.15, 33.7, 28.0, 25.3, 21.8, 14.0. The data is consistent with the literature .[(14)]

**Ethyl 4-((4*S*,5*S*)-5-formyl-2,2-dimethyl-1,3-dioxolan-4-yl) butanoate (7)**

The alcohol 6 (0.2 g, 0.8126) was dissolved in 10 ml DCM and (0.45g, 2.82 mmol) and $SO_3$-Pyr was added to the solution at 0°C under $N_2$. A solution of triethylamine (0.40 g, 0.55 ml) in DMSO (2 mL) was stirring for 5 min before being added to the reaction mixture at 0°C. The reaction mixture was left stirring for 1h at room temperature. The reaction was monitored by TLC, product is UV active. After completion the reaction was quenched by distilled water (10 mL) and stirred for 5 min before work up. The aqueous phase was extracted with DCM and the organic phase was concentrated *in vacuo*. The crude was washed with water (2*20 mL), sodium bicarbonate (3*20 mL) and brine (3*20 mL), dried over $Na_2SO_4$, filtered and concentrated *in vacuo* affording brownish orange oil (0.173 g, 87%) ethyl 4-(5-formyl-2,2-dimethyl-1,3-dioxolan-4-yl)butanoate 7. TLC; $R_f$ = 0.12 (heptane/ethylacetate, 4:1); HRMS (ESI) m/z: $[M+Na]^+$ Calcd. For $C_{12}H_{20}O_5Na$ 267.1200, found $C_{12}H_{20}O_5Na$ 267.1200 , and $[M+MeOH]^+$ 299.1500; $^1$H-NMR ($CDCl_3$, 400 MHz) δ =9.6 (s, 1H), 4.30-4.25 (m, 1H), 4.19 (dd, *J*= 4, 16 Hz, 1H), 4.05 (q, *J*= 8, 2H), 2.26 ( t, *J*= 8Hz, 2H), 1.78-1.72( m, 1H), 1.66-1.60 (m, 1H), 1.59, 1.54 (m, 1H), 1.50 (s, 3H), 1.33 (s, 3H), 1.18 (t, *J*= 8 Hz, 3H); $^{13}$C-NMR ($CDCl_3$, 100 MHz) δ= 202.4, 173.3, 110.9, 82.3, 78.6, 60.6, 34.1, 29.4, 27.9, 25.6, 22.3, 14.3.

**1-(*p*-Tolyl)heptan-1-one (10)**

Aluminium chloride (4.42 g, 33.2 mmol) was dissolved in DCM (20 mL) at 0°C. Heptanoyl chloride (4.90 g, 32.94 mmol) was added dropwise to the reaction mixture followed by the addition of toluene (2.73 g, 3.15 mL, 29.6 mmol). The mixture continued stirring for 1h at room temperature, the reaction was monitored by TLC. Upon completion the reaction mixture was poured slowly with stirring into a baker contained 30 g of ice and conc HCl (10 mL), transferred into a separation funnel and extracted with ethyl acetate (2X30 ml). The organic phase was collected and washed with saturated sodium bicarbonate (2X20 mL), brine (2*20 mL), dried over $Na_2SO_4$ then concentrated *in vacuo*. Crystals were collected and purified by recrystallization in ethanol/water (5:1), filtered and dried with air affording white crystals of 10 (4.387 g, 73 %). m.p. : 35.5-36.8 °C. TLC; $R_f$ = 0.80 (hexan/ethyl acetate, 9:1); $^1$H-NMR ($CDCl_3$, 400 MHz) δ =7.85 (d, *J* = 8Hz, 2H), 7.24 (d, *J*= 8 Hz, 2H), 2.92 (t, *J* = 8 Hz, 2H), 2.40 (s, 3H), 1.74-1.68 (m, 3H), 1.37-1.32 (m, 7H) , 0.88 (t, *J* = 4 Hz, 3H) ;$^{13}$C NMR ($CDCl_3$, 100 MHz) δ= 200.4, 143.7, 134.8 , 129.3 ,128.3, 38.7, 31.8, 29.2, 24.6, 22.7 ,21.7, 14.2. The data is consistent with literature. [29]

**1-(4-(Bromomethyl)phenyl)heptan-1-one (11)**

The acyl toluene 10 (3 g, 14.68 mmol) was dissolved in Trifluorotoluene (12 mL). A solution of NBS (2.62 g, 14.72 mmol) in $H_2O$ (30 mL) was added dropwise to the reaction mixture. The reaction was left Stirring and illuminated with a 60 W incandescent light bulb overnight, the reaction was monitored by TLC. Crude was diluted with water and extracted with ethyl acetate (2*30 mL). The organic phase was washed with brine (2*30 mL), dried over $Na_2SO_4$, filtered, and concentrated *in vacuo*. The product was recrystallized in ethanol/water (5:1). Crystals was filtered, washed with water, and dried with air affording white crystals of 11 (3.46 g, 83 %). m.p.: 49-50 °C. TLC ; $R_f$ = 0.2 (hexane/ethyl acetate, 9:1); $^1$H-NMR ($CDCl_3$, 400 MHz) δ =7.93 (d, *J*= 8, 2H), 7.48 (d, *J*= 8, 2H), 4.50 (s, 2H), 2.95 (t, *J* = 8 Hz, 2H), 1.73 (m, 2H), 1.40-1.32 (m, 6H), 0.89 (t, *J* = 4 Hz, 3H); $^{13}$C-NMR (CDCl3, 100 MHz) δ = 200.0 ,171.3, 142.6, 137.0, 131.8, 129.3, 128.7, 127.0, 125.3, 60.5, 38.8, 32.3, 31.8, 29.2, 24.4, 22.7, 21.2, 14.2. The data is consistent with literature. [30]

**(4-Heptanoylbenzyl) triphenylphosphonium (12)**

1-(4-(bromomethyl) phenyl) heptan-1-one 11 (1.73 g, 6.1 mmol) was dissolved in acetone (50 mL) followed by addition of triphenylphosphine (1.86 g, 6.58 mmol). The reaction mixture stirred under reflux for 3h at 65°C, the reaction was monitored by TLC. The crude was cooled down in an ice bath forming crystals. The crystals were collected by filtration, rinsed with diethyl ether, and dried in a desiccator. White crystals of the Wittig salt 12 were obtained, (1.58 g, 82 %). m.p.: 237-236 °C. TLC; $R_f$ = 0.7 (hexane/ethyl acetate, 9:1); IR (cm$^{-1}$) 3044, 2925, 2852, 2771, 1679, 1436, 689; HRMS (ESI) m/z: $[M+H]^+$ Calcd. For $C_{32}H_{34}OP$ 453.2300, found 453.2300 ; $^1$H-NMR ($CDCl_3$, 400 MHz) δ =7.80-7.75 (m, 10H), 7.68 (d, *J*=8 Hz, 2H), 7.63 (dd, *J*= 4, 16 Hz, 5H), 7.60 (d, *J*=4 Hz,2H), 7.28 (d, *J*= 4 Hz, 1H) , 7.26 (d, *J*=4 Hz,1H), 5.64 (d, *J*= 16 Hz, 2H), 2.85 (t, *J* = 8 Hz, 2H), 1.66 -1.63 (m, 5H), 1.36-1.30 (m, 7H), 0.88 (t, *J*=4 Hz, 3H); $^{13}$C-NMR ($CDCl_3$, 100 MHz) δ = 200.0, 142.7, 137.0, 129.3, 128.7, 38.9, 32.3, 31.8, 29.2, 24.4, 22.7, 14.2. The data is consistent with literature. [31, 32]

**1-(Hexyloxy)-4-methylbenzene (15)**

*p*-Cresol (3 g, 2.901 mL, and 27.77 mmol) was dissolved in acetonitrile (50 mL). $K_2CO_3$ (7.6 g, 55 mmol) and 1-bromohexane (4.94 g, 4.25 mL, 30.547 mmol) were added to the reaction mixture. The mixture was refluxed at 85°C under $N_2$ gas for 24 h, reaction was monitored by TLC. Upon completion, the crude was left to cool down, gravity filtered to remove the access of the base, and rinsed with DCM. The crude was washed with 1M NaOH (2*50 mL), $H_2O$ (2*50 mL), brine (2*20 mL), the aqueous phase was extracted with DCM and dried over $Na_2SO_4$. The organic phase was filtered and finally concentrated *in vacuo* affording the pure product 15 (4.1 g, 72%). The crude was used without further purification. TLC; $R_f$ =0.5 (hexane/ ethyl acetate, 9.5: 0.5); $^1$H-NMR ($CDCl_3$, 400 MHz) δ =7.10 (d, *J*= 8 Hz, 2H), 6.83 (d, *J*= 8 Hz, 2H), 3.95 (t, *J*= 8 Hz, 2H) , 2.32 (s, 3H), 1.82-1.77 (m, 2H), 1.53-1.48 (m, 2H), 1.39-1.38 (m,4H), 0.95 (t, *J*= 8Hz, 3H); $^{13}$C-NMR ($CDCl_3$, 100 MHz) δ =157.5, 130.3, 130.0, 114.8, 68.5 , 32.1 , 29.8, 26.2, 23.1 , 20.9 , 14.5. The data is consistent with literature. [31, 32]

**1-(Bromomethyl)-4-(hexyloxy)benzene (16)**

1-(hexyloxy)-4-methylbenzene (3.5 g, 18.21 mmol) was dissolved in chloroform (50 mL), NBS (3*0.35 g, 5.95 mmol) and benzoyl peroxide (3*0.2 g) were added to the reaction mixture in three equal portions. The reaction mixture was refluxed at 68°C for 9h. Upon completion, the mixture was cooled down and filtered. Crude product was washed with saturated $NaHCO_3$ solution (2*100 mL), brine (2*100 mL), dried over $Na_2SO_4$, filtered and concentrated *in vacuo*. Purification by vacuum distillation ≈4 torr and remaining amount separated using short path distillation (kugler-distill) affording pure colourless oil (2.3 g, 48%) of 1-(bromomethyl)-4-(hexyloxy) benzene 16. $^1$H-NMR ($CDCl_3$, 400 MHz,) δ =7.10 (d, *J*= 12 Hz, 2H), 6.83 (d, *J*= 8 Hz, 2H), 5.30 (s, 2H), 3.95 (t, *J*= 4 Hz, 2H), 2.32 (s, 3H), 1.83-1.76 (m, 2H), 1.53-1.46 (m, 2H), 1.39-1.35 (m, 4H), 0.94 (t, *J*= 8 Hz, 3H);$^{13}$C-NMR ($CDCl_3$, 100 MHz) δ =157.1, 129.9, 114.5, ,68.2, 53.5, 31.7, 29.4, 25.9, 22.7, 20.5, 14.1. The data is consistent with literature. [32]

**(4-(Hexyloxy) benzyl) triphenylphosphonium (17)**

The ether **16** (0.565g, 2.07mmol) was dissolved in acetone (20 mL), triphenylphosphine (0.58 g, 2.25 mmol) was added to the reaction mixture and stirred for 3h under reflux at 65°C, the reaction was monitored by TLC. Upon completion, the crude was concentrated *in vacuo*, washed with ethyl acetate and filtered affording **17 as** white powder (0.676 g, 72%) **m.p.**: 360-

361°C. TLC: $R_f$ = 0.2 (hexane/ethyl acetate, 5:1); **IR** ($cm^{-1}$) 3053, 2926, 2856, 1606, 1508, 1436, 843; **HRMS** (ESI) m/z: $[M+H]^+$ Calcd. For $C_{31}H_{34}OP$ 465.2300, found 465.2300 ; **$^1$H NMR** ($CDCl_3$, 400 MHz) δ =77.74 (dt, *J* = 12.4, 6.3 Hz, 6H), 7.69 (d, *J* = 1.4 Hz, 2H), 7.63 (dd, *J* = 7.5, 3.5 Hz, 5H), 7.60 (d, *J* = 3.5 Hz, 1H), 6.98 (dd, *J*= 4, 16 Hz, 2H), 6.63(d, *J*=8 Hz, 2H), 5.29 (d, *J*= 16 Hz, 2H), 3.84 (t, *J*= 8Hz, 2H), 1.75-1.68 (m, 2H), 1.42-1.38 (m, 2H), 1.32 -1.30 (m, 4H), 0.88 (t, *J*= 8 Hz, 3H);**$^{13}$C NMR** ($CDCl_3$, 100MHz) δ =159.4, 135.0, 134.6, 134.6, 132.8, 130.2, 118.6, 117.7, 114.9, 68.2, 31.7, 29.3, 25.8, 22.7, 14.2. The data is consistent with literature. [31, 32]

**Ethyl 4-((4*S*,5*R*)-5-((*E*)-4-heptanoylstyryl)-2,2-dimethyl-1,3-dioxolan-4-yl) butanoate (18)**

The Wittig salt **12** (0.79 g, 1.44 mmol) was dissolved in THF (1 mL) at 0 °C under $N_2$ gas. A solution of Potassium t-butoxide (0.3 g, 2.7 mmol) in THF (0.5 mL) was added slowly during 15 min to the reaction mixture at 0°C. The reaction mixture was continued stirring for 20 min at room temperature. A solution of the aldehyde **7** (0. 27g, 1.11 mmol) in THF was added slowly to the reaction mixture at room temperature. After complete addition the reaction mixture continued stirring for 1-2 hr, reaction progress was monitored by TLC. Upon completion, the crude was concentrated *in vacuo*, washed with water and extracted with ethyl acetate. The organic phase was collected, dried over $Na_2SO_4$, and concentrated *in vacuo*. The crude was further purified by column chromatography (hexane, ethyl acetate, 10:1) affording the alkene **18** as yellow liquid (0.45 g, 60%). TLC; $R_f$ =0.35 (hexane/ethyl acetate, 5:1); **HRMS** (ESI) m/z: $[M+Na]^+$ Calcd. For $C_{26}H_{38}O_5Na$ 453.26, found 453.2610; **IR** ($cm^{-1}$) 2955, 2865, 1732, 1682, 1218, 1030, 856; **$^1$H-NMR** ($CDCl_3$, 400 MHz) δ =7.92 (d , *J*= 8 Hz, 2H),7.30 (d, *J*= 8 Hz, 1H) 7.71 (d , *J*= 8 Hz, 1H), 5.81 (m, 1H), 4.87 (dd, *J* = 9.7, 6.1 Hz, 1H), 4.19-4.14 (m, 1H), 4.09 (q, *J*= 8 Hz, 3H) 2.93 (t, *J*= 8 Hz, 2H), 2.33 (t, *J*= 8 Hz, 2H), 2.01 (s, 2H), 1.75-1.67 (m, 2H), 1.66-1.58 (m, 2H), 1.48 (s, 3H), 1.41 (d, *J* = 9.5 Hz, 3H), 1.38-1.29(m, 8H), 1.25-1.18 (m, 5H), 0.89 (t, *J*= 8 Hz, 3H); **$^{13}$C-NMR** ($CDCl_3$, 100MHz) δ= 200.4, 173.7, 141.1, 136.3, 132.9, 130.0, 129.2, 128.5, 108.9, 78.6, 77.8, 77.2, 74.5, 60.7, 39.0, 34.5, 32.1, 30.3, 29.5, 28.8, 26.1, 24.8, 22.9, 22.3, 14.7.

**Ethyl 4-((4*S*,5*R*)-5-((*E*)-4-(hexyloxy)styryl)-2,2-dimethyl-1,3-dioxolan-4-yl)butanoate (19)**

The Wittig salt 17 (0.2g, 0.44 mmol) was dissolved in THF (1 mL) at 0 °C under $N_2$ gas. A solution of Potassium t-butoxide (0.11 g, 0.90 mmol) in THF (0.5 mL) was added slowly during 15 min to the reaction mixture at 0°C. The reaction mixture was continued stirring for 20 min at room temperature. A solution of the aldehyde 7 (0. 1 g, 0.40 mmol) in THF (1 mL) was added slowly to the reaction mixture at room temperature. The reaction mixture left stirring for 1-2 hr, the reaction was monitored by TLC. Upon completion, the crude was concentrated *in vacuo*, washed with water and extracted with ethyl acetate. The organic phase was collected, dried over $Na_2SO_4$, and concentrated *in vacuo*. The crude was further purified by column chromatography (pentane, ethyl acetate, 9:1) affording the alkene 19 as yellow oil (0.11 g, 61%) of. TLC; Rf =0.36 (hexane/ethyl acetate, 5:1); IR ($cm^{-1}$) 2933, 1733, 1510, 1174, 1025, 839; HRMS (ESI) m/z: $[M+Na]^+$ Calcd. for $C_{25}H_{38}O_5Na$ 441.26 , found 441.2600; $^1$H-NMR ($CDCl_3$,400 MHz) δ=7.13 (d, *J*= 8 Hz, 2H), 6.84 (d, *J*= 8 Hz, 2H), 6.36 (d, *J*= 12 Hz,1H), 5.59 (m, 1H), 4.49 (m, 1H), 4.16-4.14 (m, 2H), 4.09 (q, *J* = 7.1 Hz, 5H), 3.93 (t, *J*= 8 Hz 2H), 2.32 (t, *J*= 12 Hz, 2H), 2.01 (s, 5H), 1.77-1.71 (m, 3H), 1.63-1.56 (m, 2H), 1.47 (s, 3H), 1.45-1.40 (m, 3H), 1.32 (s, 6H), 1.24-1.19 (m, 8H), 0.88 (t, *J*= 8 Hz, 3H); $^{13}$C-NMR ($CDCl_3$, 100 MHz) δ=173.4, 171.1, 158.7, 133.2, 129.9, 128.6, 125.9, 114.4, 108.2, 78.3, 74.4, 68.1, 60.4, 34.2, 31.6, 30.1, 29.3, 28.5, 25.8, 22.7, 21.9, 21.1, 14.1.

**(*S*)-6-((*R*, *E*)-3-(4-heptanoylphenyl)-1-hydroxyallyl)tetrahydro-2*H*-pyran-2-one (1)**

The alkene **18** (0.022 g, 0.05 mmol) was dissolved in a mixture of acetonitrile and water (1:1). TFA (0.8 ml) was added dropwise to the reaction mixture at 0 °C. After complete addition the ice bath was removed and the reaction kept stirring at room temperature for 1h, the reaction was monitored by TLC. Upon completion, the reaction was slowly quenched with $NaHCO_3$ and extracted several times with ethyl acetate. The combined organic phase was washed with water and brine, dried over $Na_2SO_4$ and concentrated *in vacuo* affording the lactone **1** as a colourless oil (0.012, 70 %). TLC; $R_f$ =0.45(DCM/MeOH, 9.8:0.2); **IR** ($cm^{-1}$) 2928, 2857, 2248, 2159, 1729, 1679, 1603, 1239, 1049; **HRMS** (ESI) m/z: $[M+Na]^+$ Calcd. for $C_{21}H_{28}O_4Na$ 367.1900, found 367.1883; **$^1$H-NMR** (400 MHz, $CDCl_3$) δ=7.94-7.91 (m, 2H), 7.42 (dd, *J*=22, 8 Hz, 2H), 6.74 (t, *J*=8 Hz, 1H), 5.89-5.79 (m, 1H), 4.46-4.40 (m, 1H), 4.32-4.27 (m, 1H), 3.50 (bs, 1H), 2.94 ( t, *J*=8 Hz, 2H), 2.63-2.55 (m, 1H), 2.49-2.37 (m, 1H), 1.93-1.82 (m, 2H), 1.75-1.68 (m,

3H), 1.37-1.24 (m, 7H), 0.88 (t, *J*=8 Hz, 3H); **$^{13}$C-NMR** ($CDCl_3$, 100 MHz) δ=200.3, 171.5, 171.2, 140.7, 136.2, 133.9, 133.4, 129.8, 129.0, 128.4, 83.2, 82.9, 69.8, 69.2, 38.8, 31.8, 29.8, 29.2, 24.5, 24.1, 22.6, 18.5, 18.4, 14.2.

**(*S*)-6-((*R*, *E*)-3-(4-(hexyloxy)phenyl)-1-hydroxyallyl)tetrahydro-2*H*-pyran-2-one (2)**

The alkene **19** (0.022 g, 0.05 mmol) was dissolved in a mixture of acetonitrile and water (1:1) at 0 °C. TFA (0.8 mL) was added dropwise to the stirring mixture at 0 °C. After complete addition the ice bath was removed and the reaction kept stirring at room temperature for 1h, the reaction was monitored by TLC. Upon completion, the reaction was slowly quenched with $NaHCO_3$ and extracted several times with ethyl acetate. The combined organic phase was washed with water and brine, dried over $Na_2SO_4$ and concentrated *in vacuo* affording the lactone **2** as colourless oil (0.013, 75 %). TLC; $R_f$ =0.45(DCM/MeOH, 9.8:0.2); **IR** ($cm^{-1}$) 2927, 2857, 1711, 1606, 1510, 1174, 1049, 839; **HRMS** (ESI) m/z: $[M+Na]^+$ Calcd. for $C_{20}H_{28}O_4Na$ 355.1900 found 355.1884 ; **$^{1}$H-NMR** ($CDCl_3$, 400 MHz) δ= 7.13 (d, *J*= 8 Hz, 1H), 6.84 (d, *J*= 12 Hz, 2H), 6.63 (d, *J*= 12 Hz, 1H), 5.62-5.57 (m, 1H), 4.97-4.93 (m, 1H), 4.18-4.14 (m, 1H), 4.07 (q, *J*= 8 Hz, 2H), 3.93 (t, *J*= 8 Hz, 2H), 2.32 (m, 2H), 1.79-1.62 (m, 3H), 1.66-1.56 (m, 2H), 1.47 (s, 3H), 1.45-1.41 (m, 2H), 1.32 (s, 6H), 1.23-1.20 (m, 3H), 0.88 ( t, *J*= 8 Hz, 3H) ; **$^{13}$C-NMR** ($CDCl_3$, 100 MHz,) δ= 173.9, 159.1, 133.7, 130.3, 129.0, 126.2, 114.8, 108.6, 78.7, 74.8, 68.5, 60.7, 34.6, 32.1, 30.6, 29.7, 28.9, 22.3, 14.5.

# Spectroscopic data

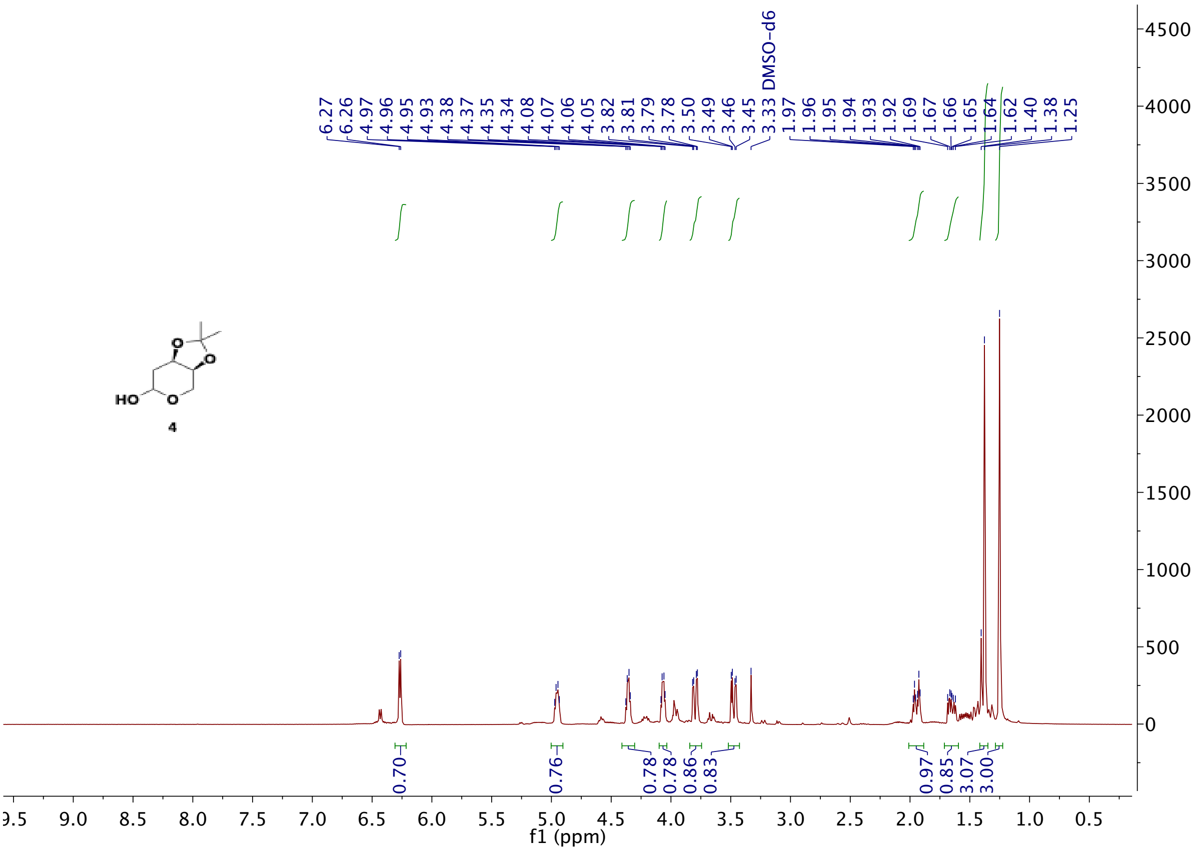


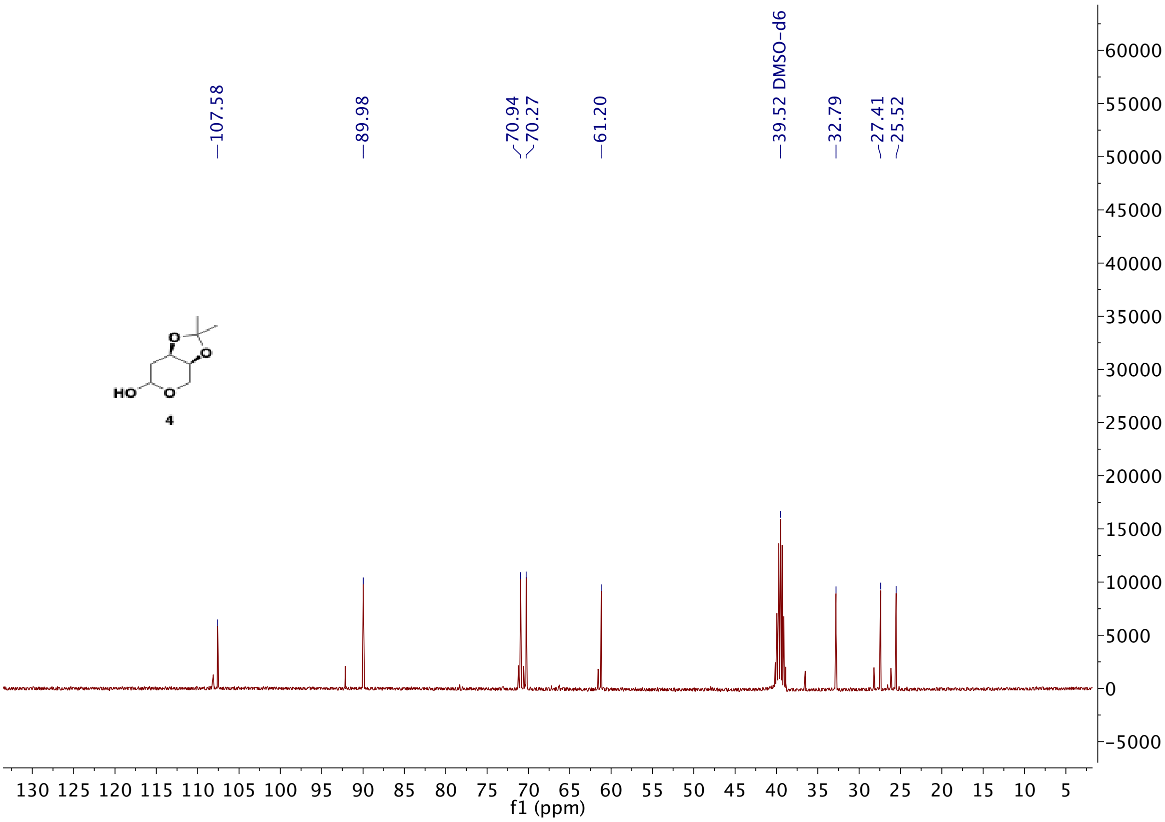

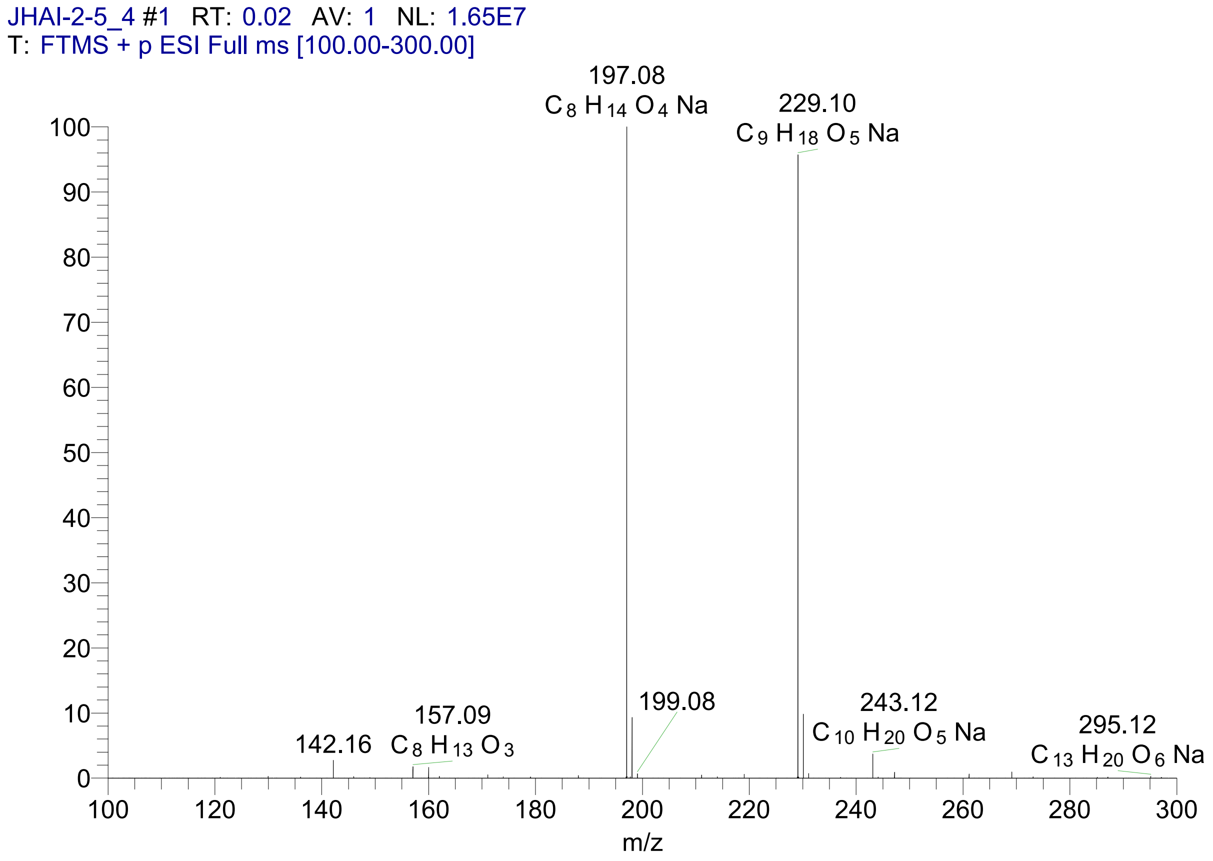
JHAI-2-5_4 #1 RT: 0.02 AV: 1 NL: 1.65E7
T: FTMS + p ESI Full ms [100.00-300.00]
197.08
C 8 H 14 O 4 Na
229.10
C 9 H 18 O 5 Na
199.08
243.12
C 10 H 20 O 5 Na
157.09
C 8 H 13 O 3
142.16
295.12
C 13 H 20 O 6 Na
100
90
80
70
60
50
40
30
20
10
0
100
120
140
160
180
200
220
240
260
280
300
m/z

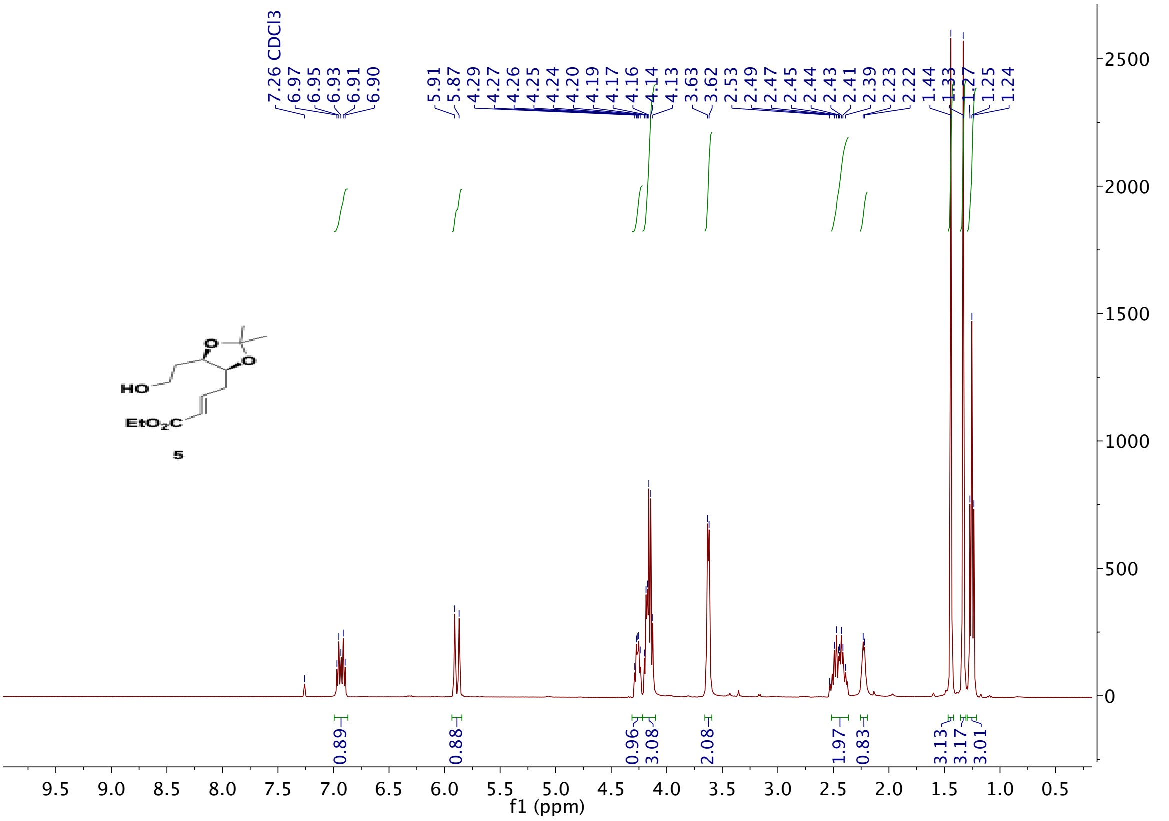
7.26 CDCl3
6.97
6.95
6.93
6.91
6.90
5.91
5.87
4.29
4.27
4.26
4.25
4.24
4.20
4.19
4.17
4.16
4.14
4.13
3.63
3.62
2.53
2.49
2.47
2.45
2.44
2.43
2.41
2.39
2.23
2.22
1.44
1.33
1.27
1.25
1.24
HO
EtO2C
5
0.89
0.88
0.96
3.08
2.08
1.97
0.83
3.13
3.17
3.01
f1 (ppm)

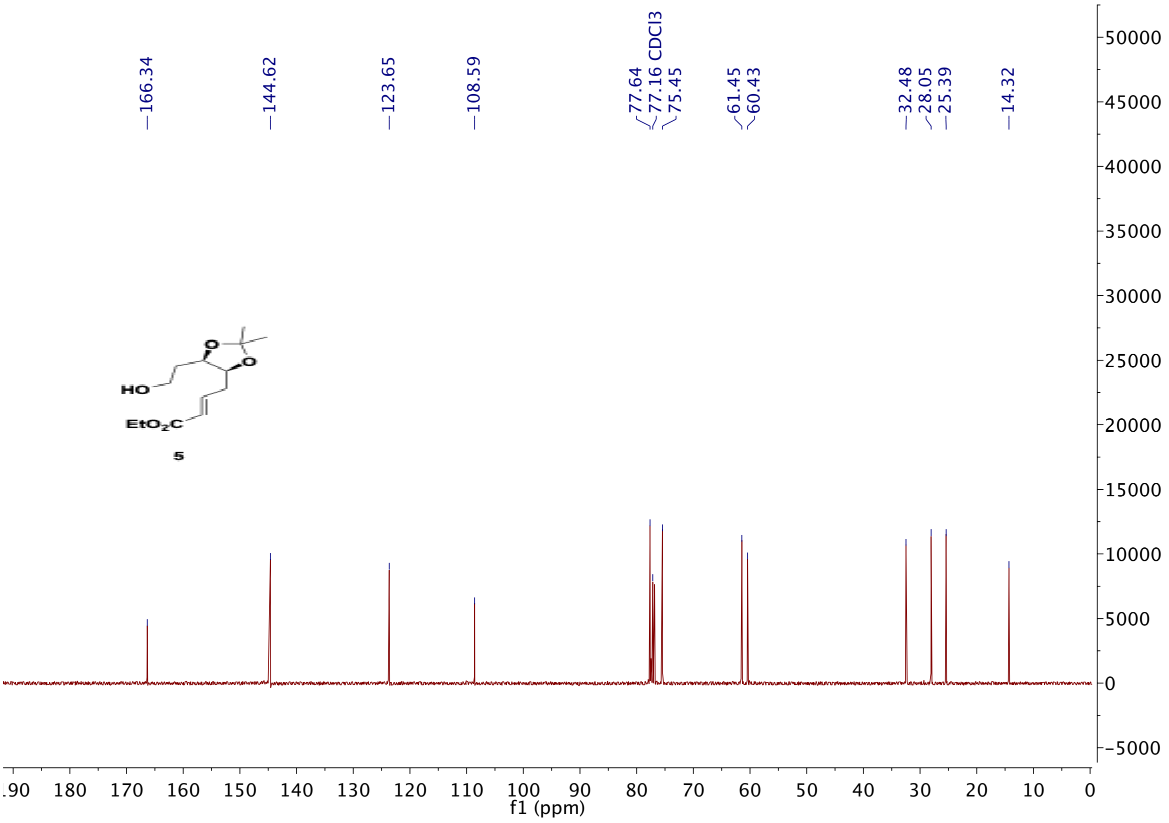
166.34
144.62
123.65
108.59
77.64
77.16 CDCl3
75.45
61.45
60.43
32.48
28.05
25.39
14.32
HO
EtO2C
5
f1 (ppm)

UNALK_151002103248 #1 RT: 0.01 AV: 1 N E7
T: FTMS + p ESI Full ms [200.00-500.00]

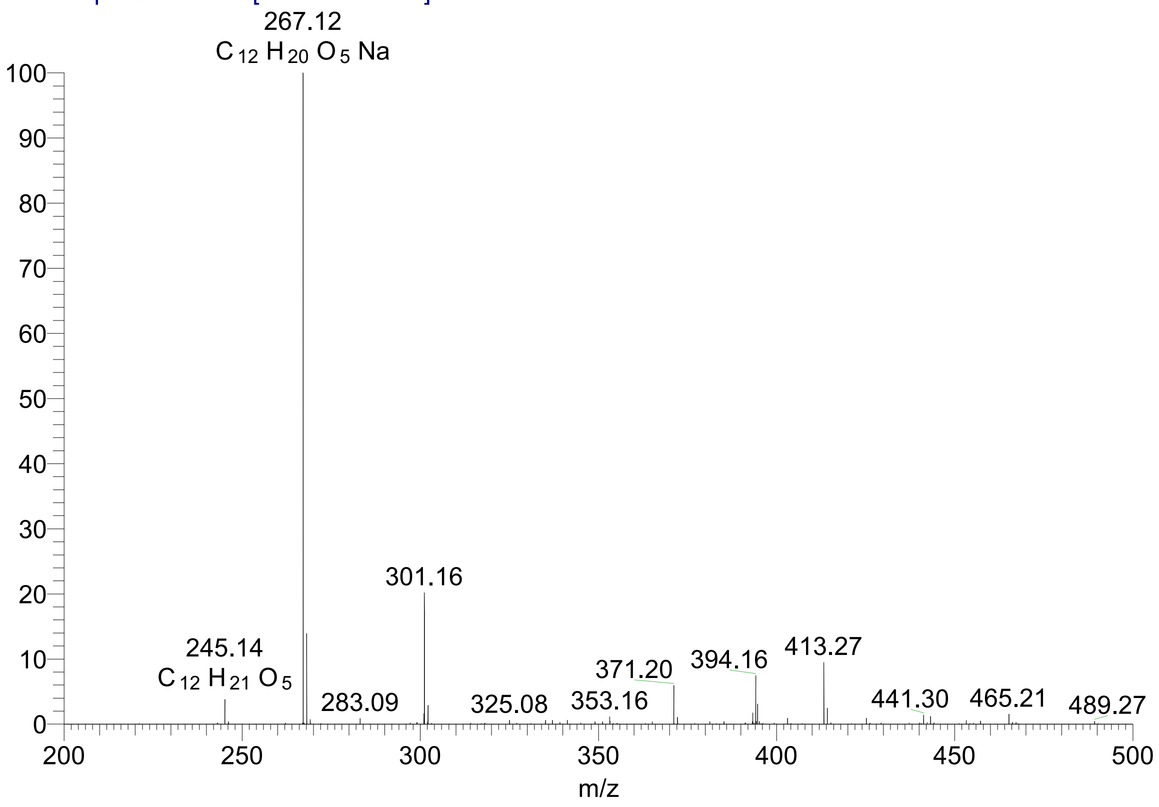

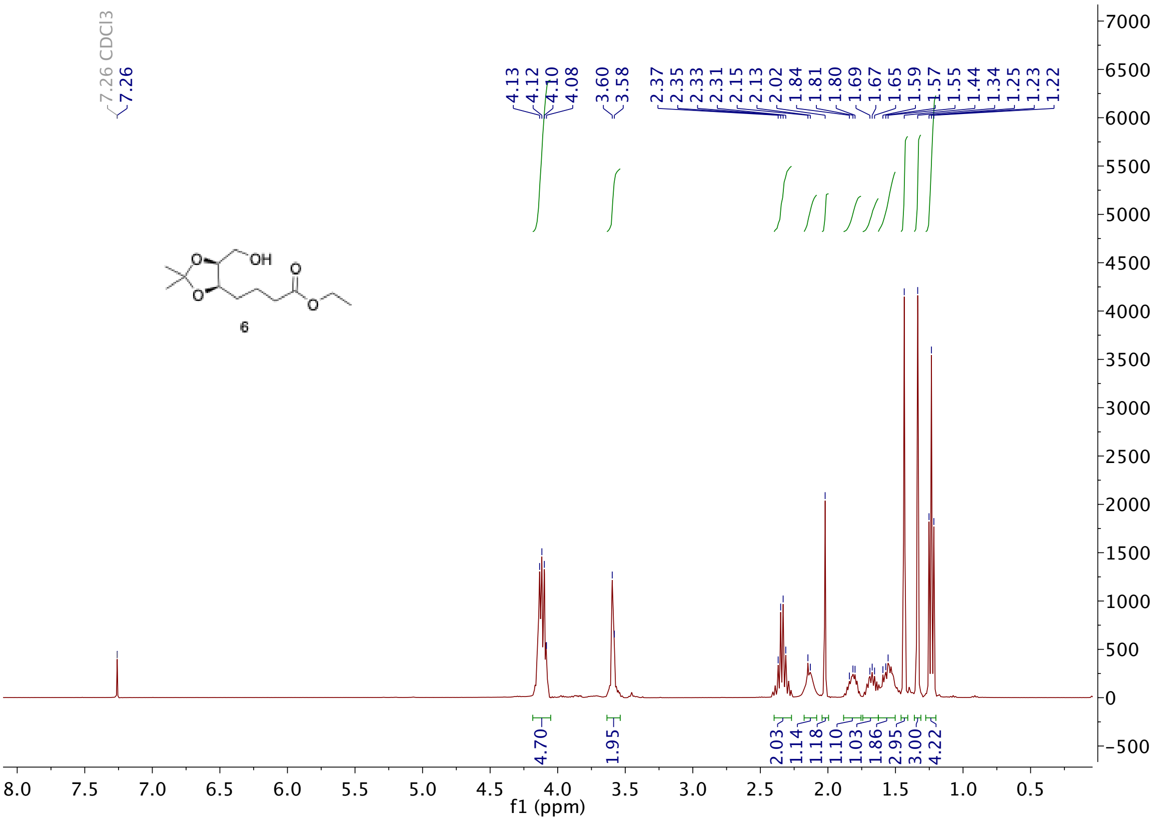
7.26 CDCl3
7.26
4.13
4.12
4.10
4.08
3.60
3.58
2.37
2.35
2.33
2.31
2.15
2.13
2.02
1.84
1.81
1.80
1.69
1.67
1.65
1.59
1.57
1.55
1.44
1.34
1.25
1.23
1.22
OH
O
O
O
O
6
4.70
1.95
2.03
1.14
1.18
1.10
1.03
1.86
2.95
3.00
4.22
f1 (ppm)

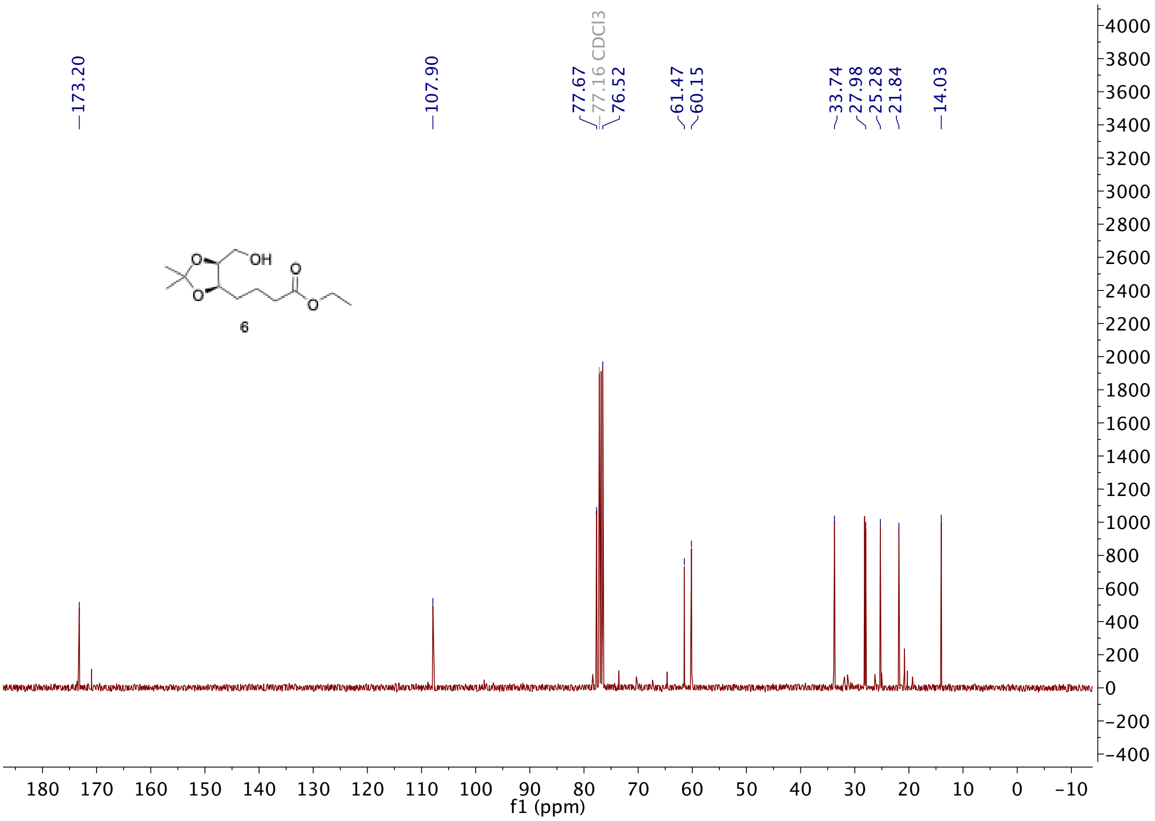
173.20
107.90
77.67
77.16 CDCl3
76.52
61.47
60.15
33.74
27.98
25.28
21.84
14.03
OH
O
O
O
O
6
f1 (ppm)

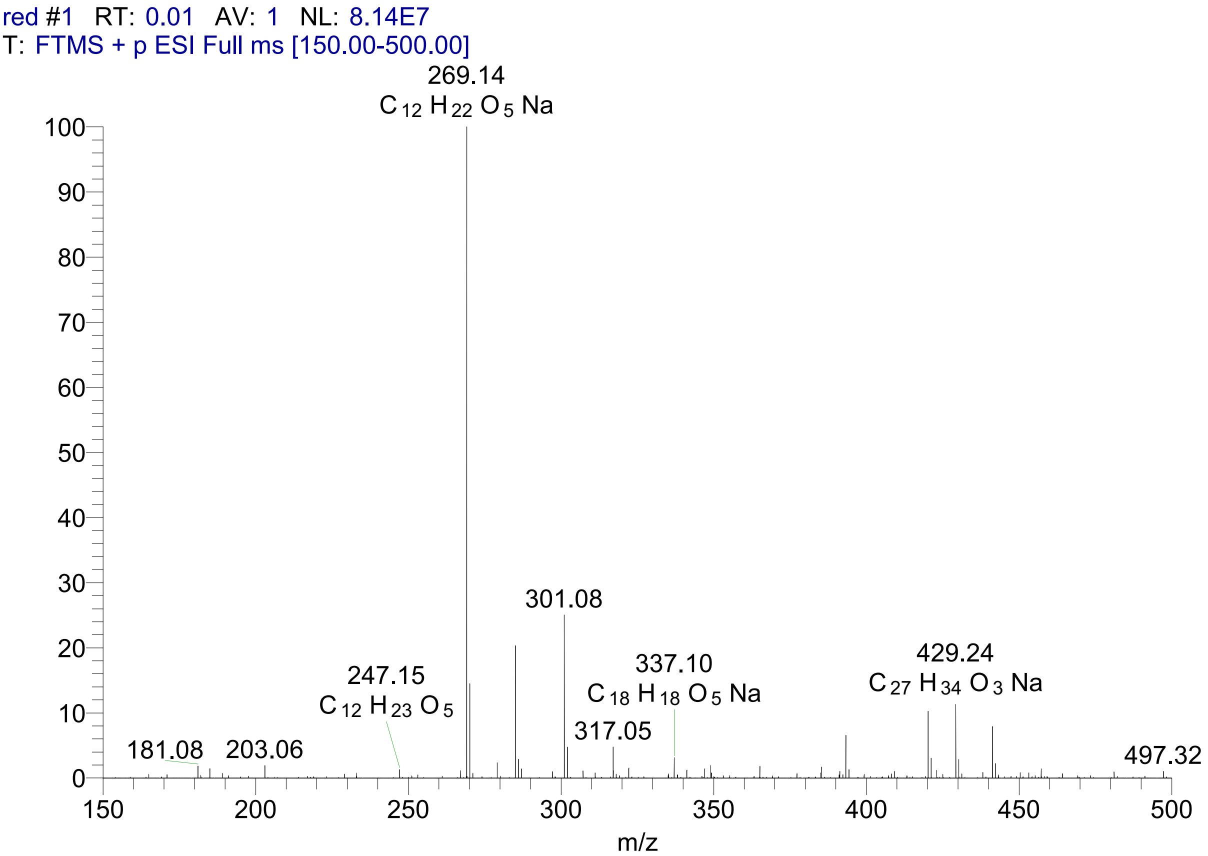
red #1 RT: 0.01 AV: 1 NL: 8.14E7
T: FTMS + p ESI Full ms [150.00-500.00]
269.14
C 12 H 22 O 5 Na
301.08
337.10
C 18 H 18 O 5 Na
429.24
C 27 H 34 O 3 Na
247.15
C 12 H 23 O 5
317.05
181.08
203.06
497.32
m/z

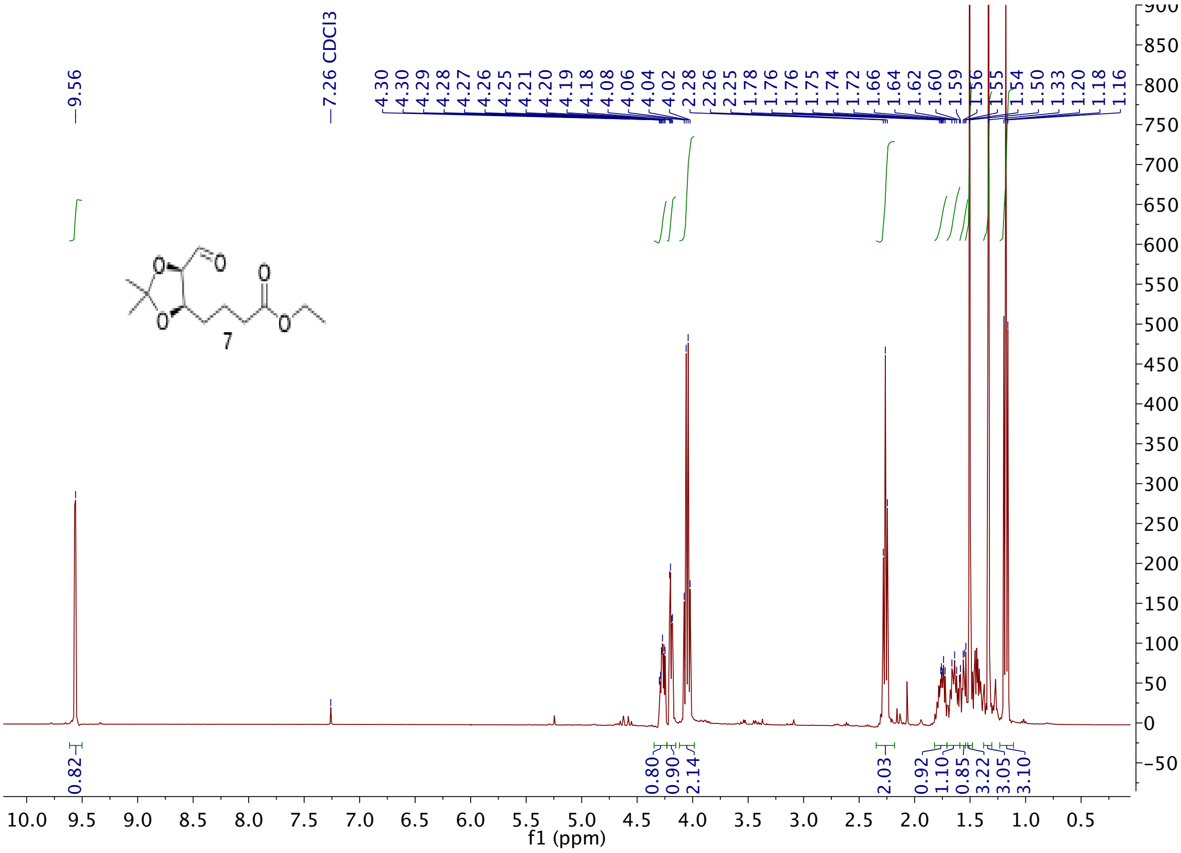
9.56
7.26 CDCl3
4.30 4.30 4.29 4.28 4.27 4.26 4.25 4.21 4.20 4.19 4.18 4.08 4.06 4.04 4.02 2.28 2.26 2.25 1.78 1.76 1.76 1.75 1.74 1.72 1.66 1.64 1.62 1.60 1.59 1.56 1.55 1.54 1.50 1.33 1.20 1.18 1.16
7
0.82
0.80 0.90 2.14
2.03
0.92 1.10 0.85 3.22 3.05 3.10
f1 (ppm)

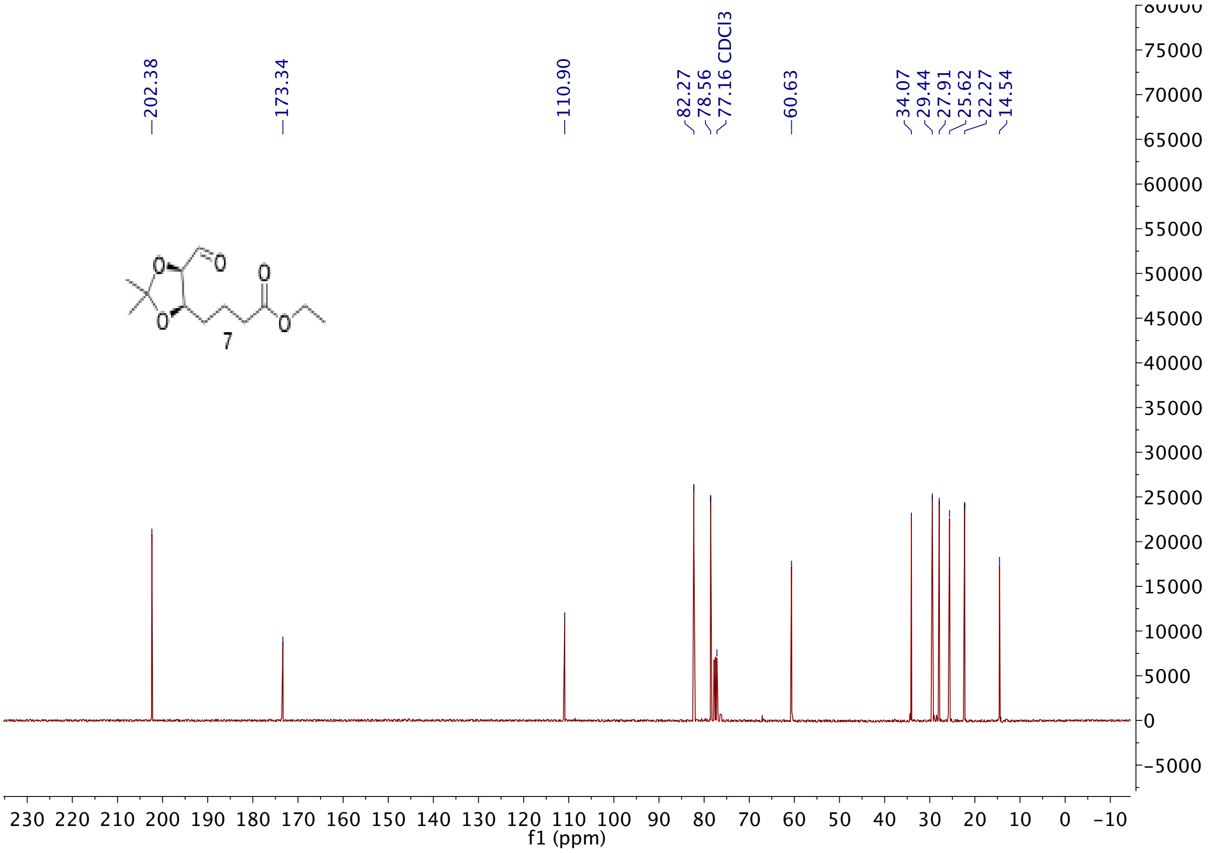

202.38
173.34
110.90
82.27
78.56
77.16 CDCl3
60.63
34.07
29.44
27.91
25.62
22.27
14.54
f1 (ppm)


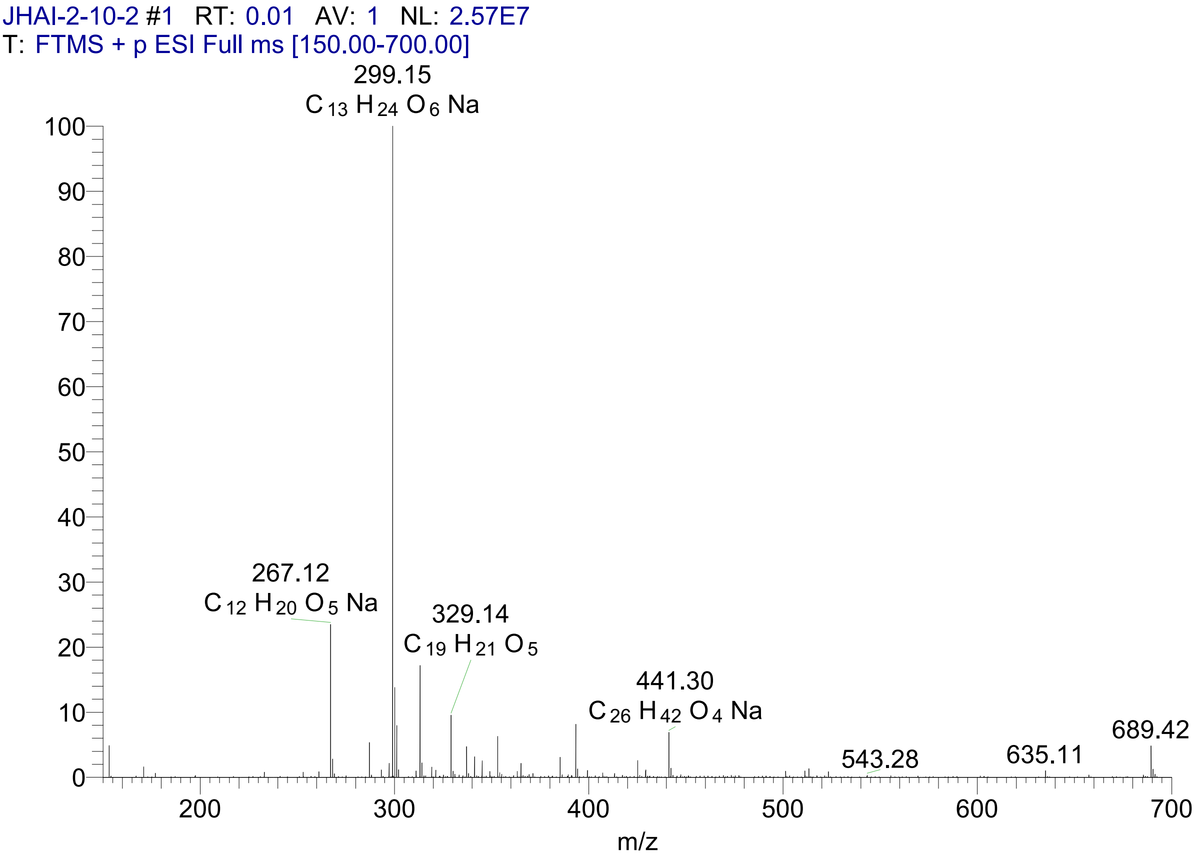

JHAI-2-10-2 #1 RT: 0.01 AV: 1 NL: 2.57E7
T: FTMS + p ESI Full ms [150.00-700.00]
299.15
C 13 H 24 O 6 Na
267.12
C 12 H 20 O 5 Na
329.14
C 19 H 21 O 5
441.30
C 26 H 42 O 4 Na
543.28
635.11
689.42
m/z

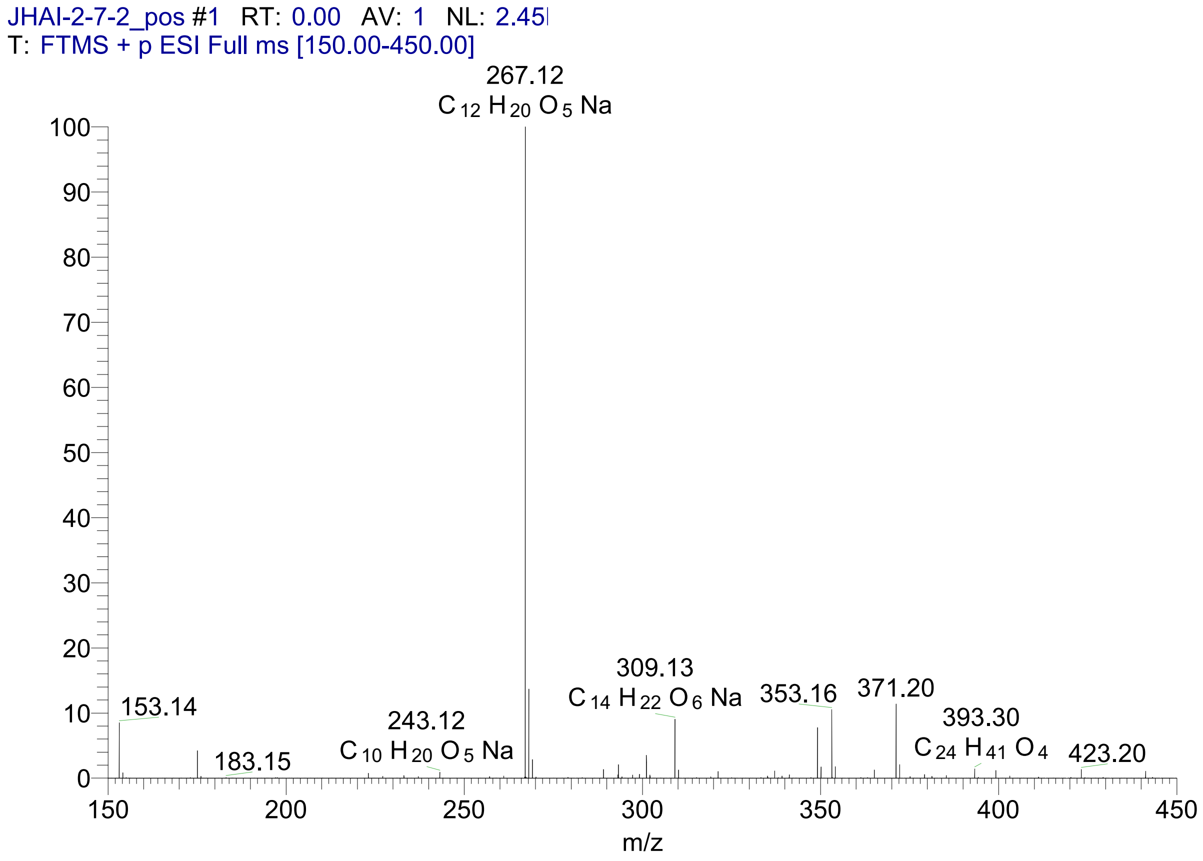
JHAI-2-7-2_pos #1 RT: 0.00 AV: 1 NL: 2.45
T: FTMS + p ESI Full ms [150.00-450.00]
267.12
C 12 H 20 O 5 Na
153.14
183.15
243.12
C 10 H 20 O 5 Na
309.13
C 14 H 22 O 6 Na
353.16
371.20
393.30
C 24 H 41 O 4
423.20
m/z

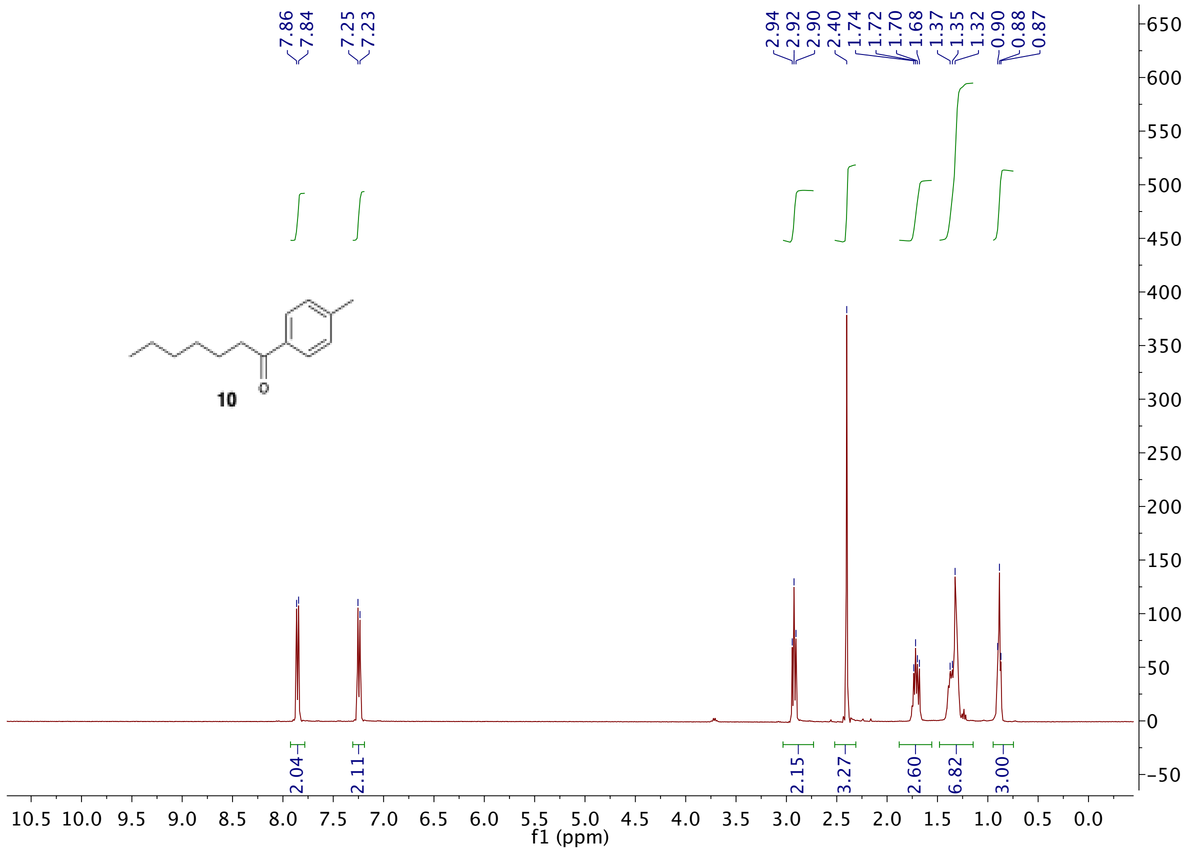
7.86
7.84
7.25
7.23
2.94
2.92
2.90
2.40
1.74
1.72
1.70
1.68
1.37
1.35
1.32
0.90
0.88
0.87
10
2.04
2.11
2.15
3.27
2.60
6.82
3.00
f1 (ppm)

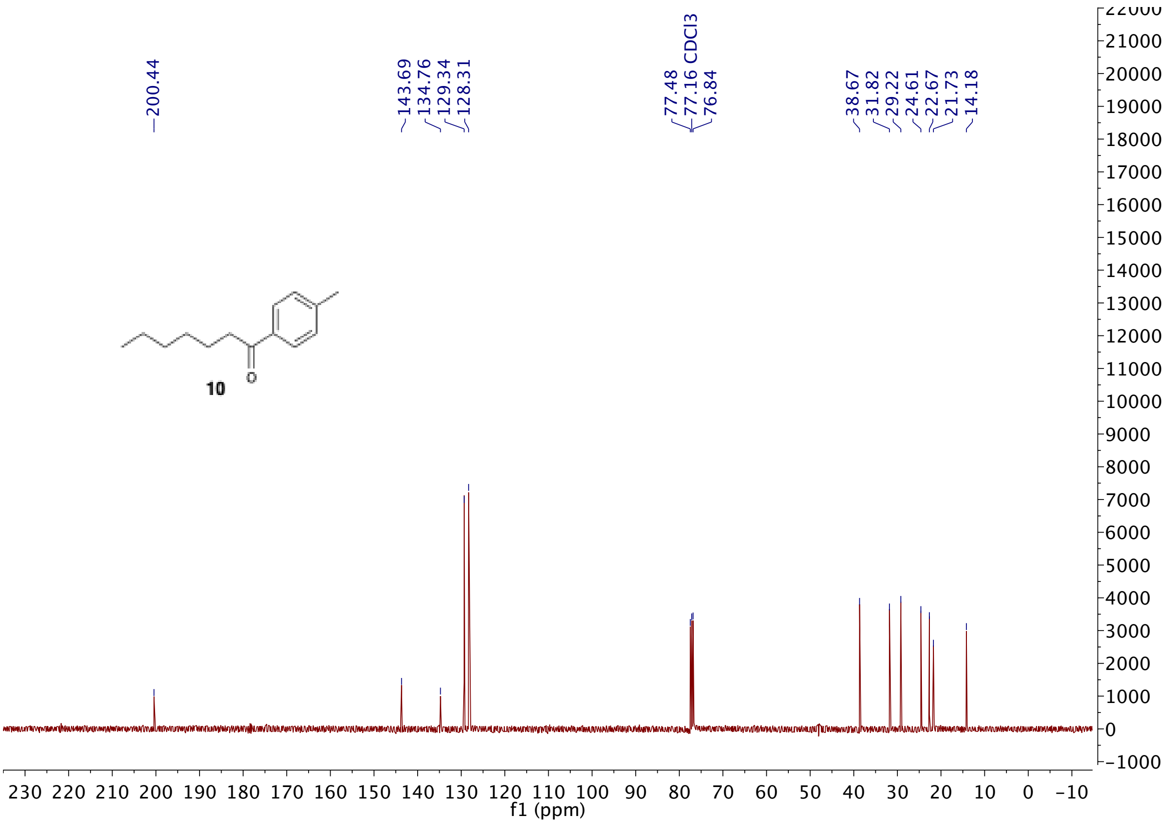
200.44
143.69
134.76
129.34
128.31
77.48
77.16 CDCl3
76.84
38.67
31.82
29.22
24.61
22.67
21.73
14.18
10
f1 (ppm)

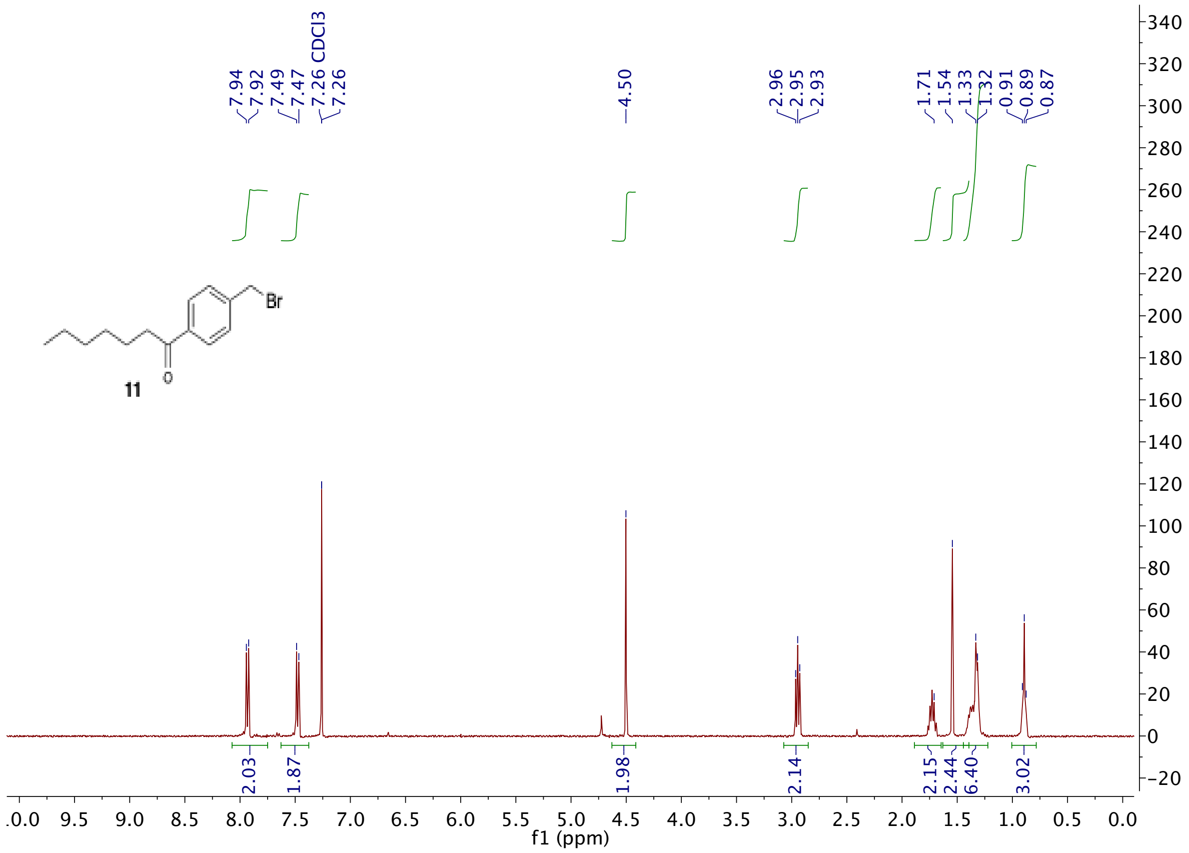
7.94
7.92
7.49
7.47
7.26 CDCl3
7.26
4.50
2.96
2.95
2.93
1.71
1.54
1.33
1.32
0.91
0.89
0.87
Br
11
2.03
1.87
1.98
2.14
2.15
2.44
6.40
3.02
f1 (ppm)

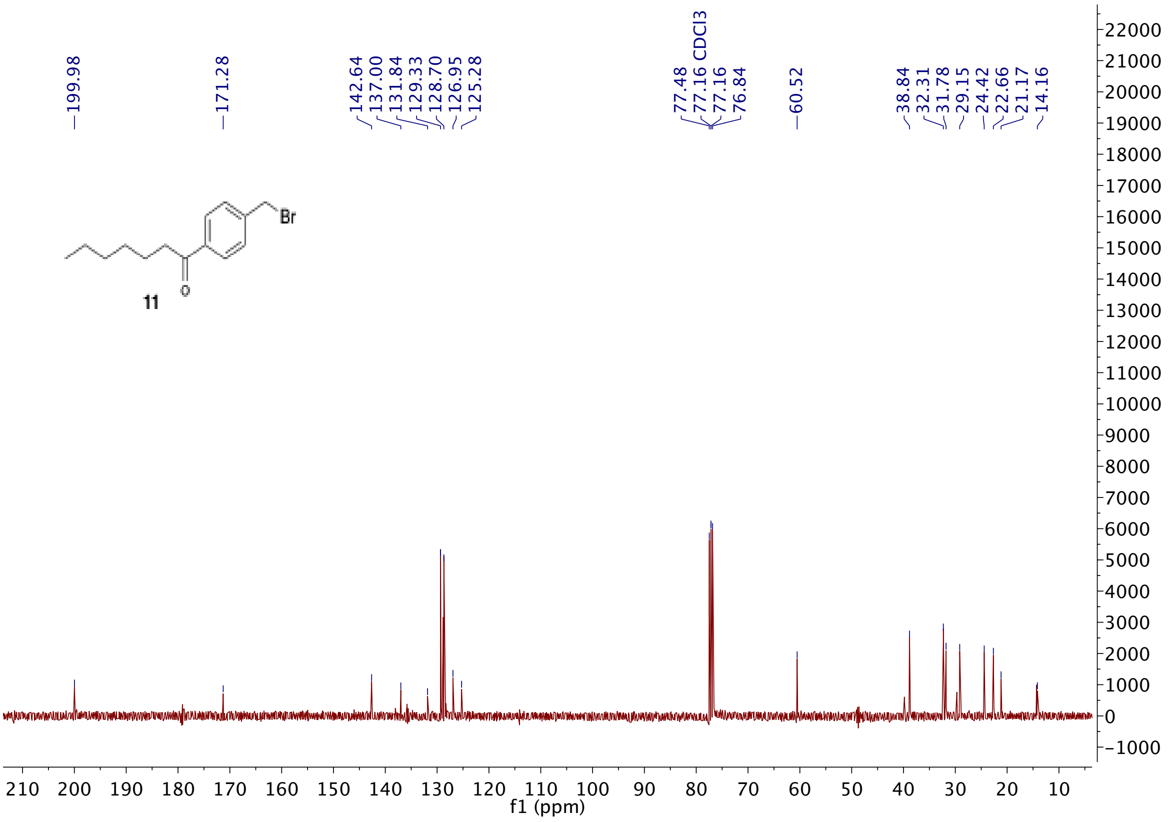

199.98
171.28
142.64
137.00
131.84
129.33
128.70
126.95
125.28
77.48
77.16 CDCl3
77.16
76.84
60.52
38.84
32.31
31.78
29.15
24.42
22.66
21.17
14.16
Br
O
11
f1 (ppm)


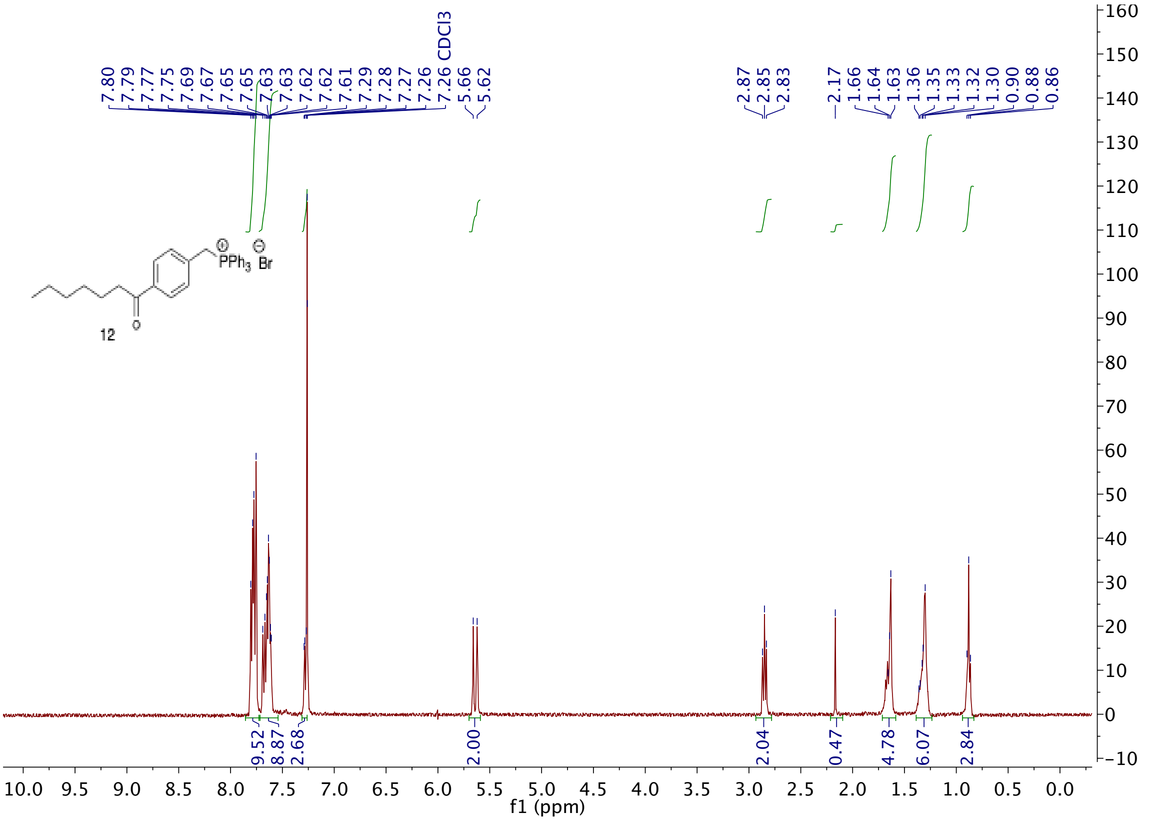

7.80
7.79
7.77
7.75
7.69
7.67
7.65
7.63
7.63
7.62
7.62
7.61
7.29
7.28
7.27
7.26
7.26 CDCl3
5.66
5.62
2.87
2.85
2.83
2.17
1.66
1.64
1.63
1.36
1.35
1.33
1.32
1.30
0.90
0.88
0.86
PPh3
Br
O
12
9.52
8.87
2.68
2.00
2.04
0.47
4.78
6.07
2.84
f1 (ppm)

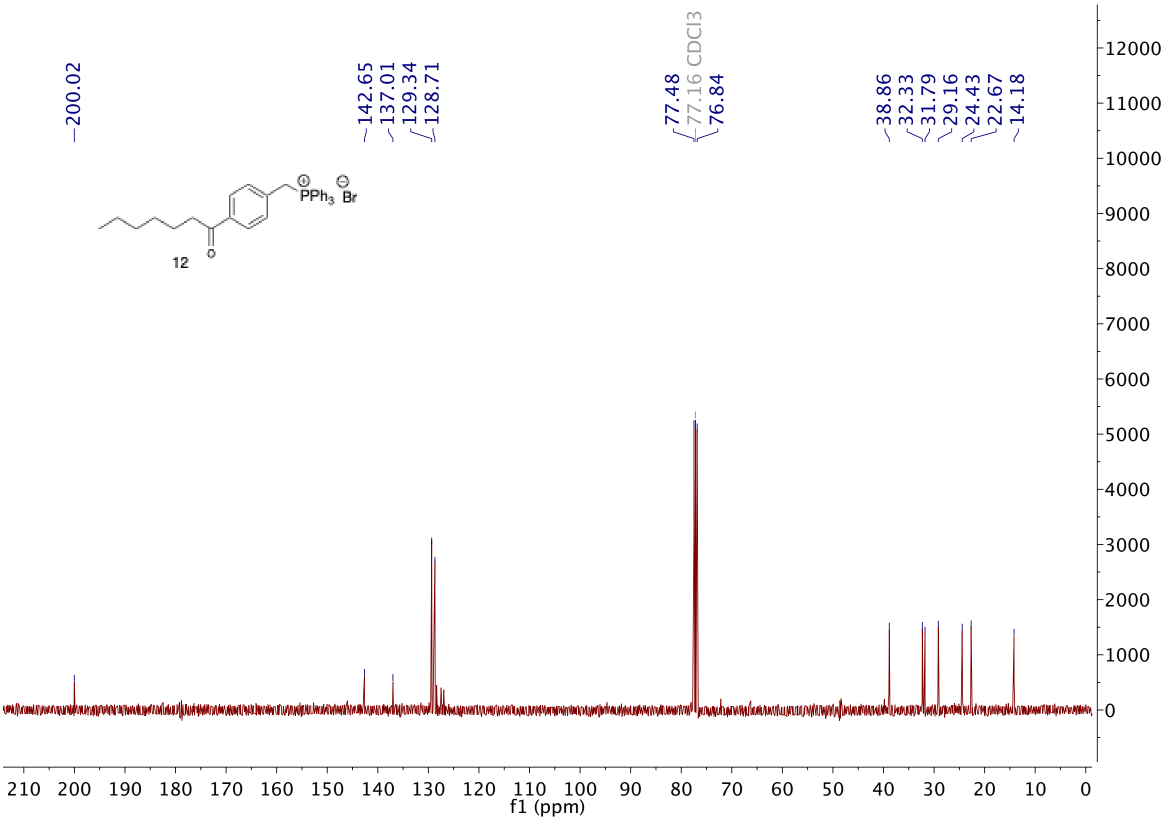
200.02
142.65
137.01
129.34
128.71
77.48
77.16 CDCl3
76.84
38.86
32.33
31.79
29.16
24.43
22.67
14.18
PPh3
Br
12
f1 (ppm)

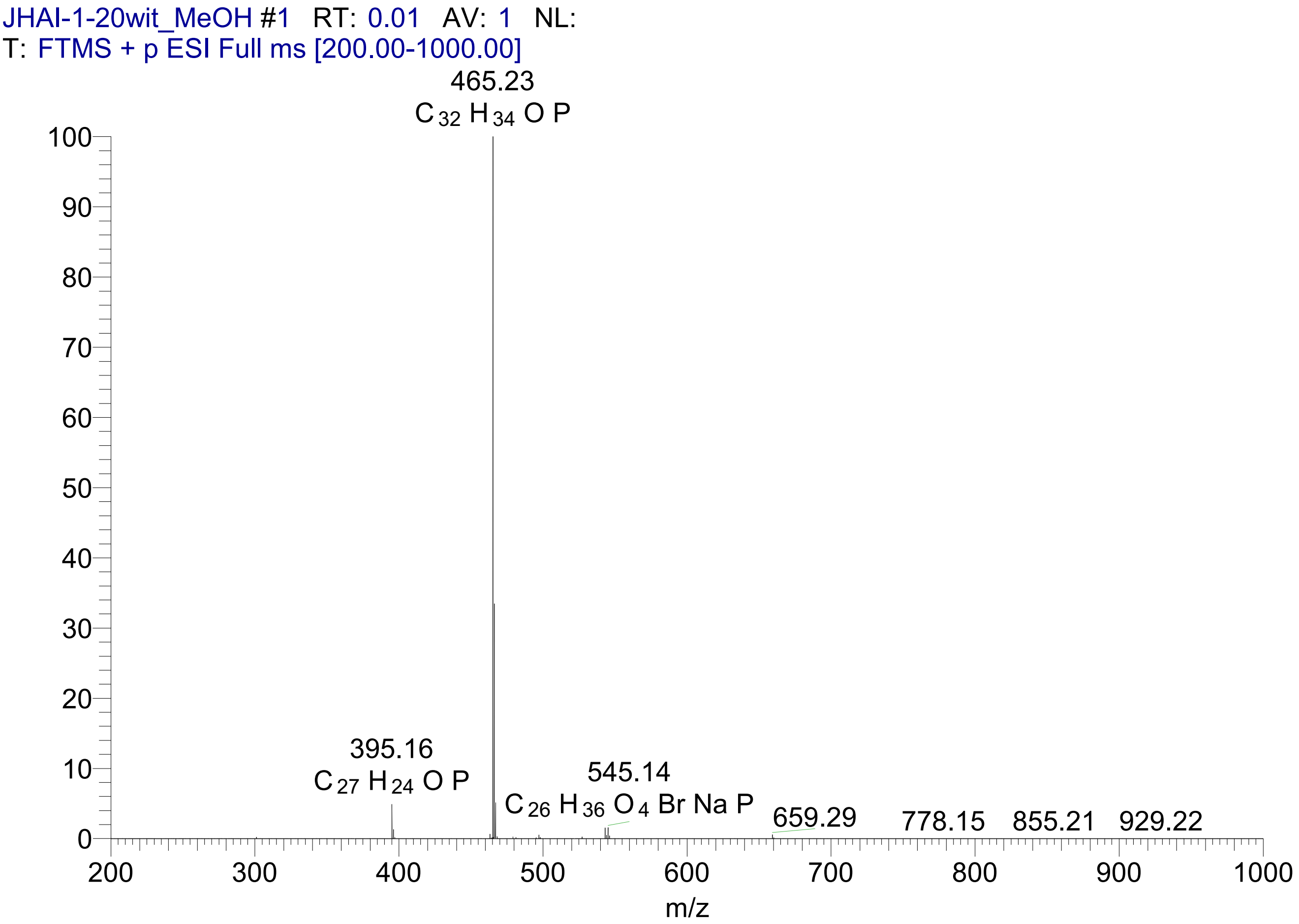
JHAI-1-20wit_MeOH #1 RT: 0.01 AV: 1 NL:
T: FTMS + p ESI Full ms [200.00-1000.00]
465.23
C 32 H 34 O P
395.16
C 27 H 24 O P
545.14
C 26 H 36 O 4 Br Na P
659.29
778.15
855.21
929.22
m/z

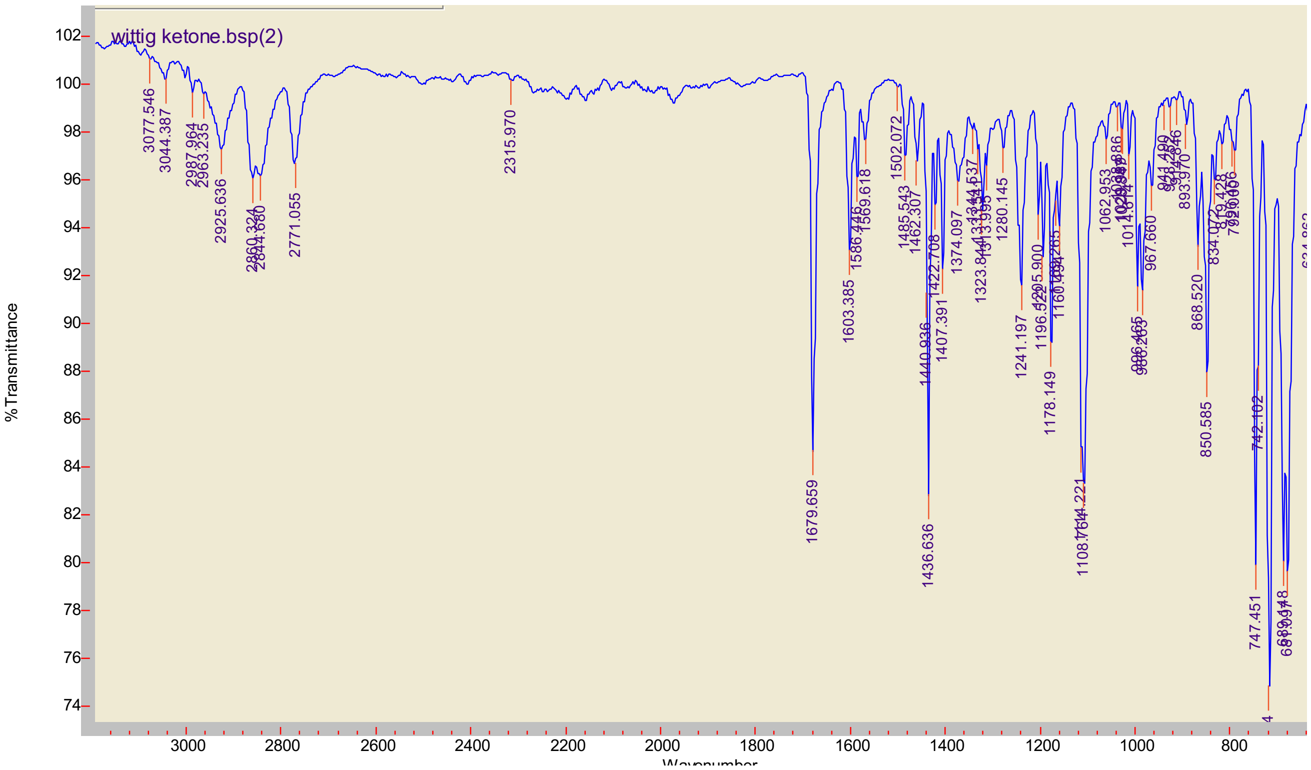
wittig ketone.bsp(2)
% Transmittance
3077.546
3044.387
2987.964
2963.235
2925.636
2771.055
2315.970
1679.659
1603.385
1569.618
1502.072
1485.543
1462.307
1436.636
1422.708
1407.391
1374.097
1323.844
1280.145
1241.197
1178.149
1108.764
1062.953
1014.674
967.660
941.490
893.970
868.520
850.585
834.072
747.451
742.102
681.931
Wavenumber

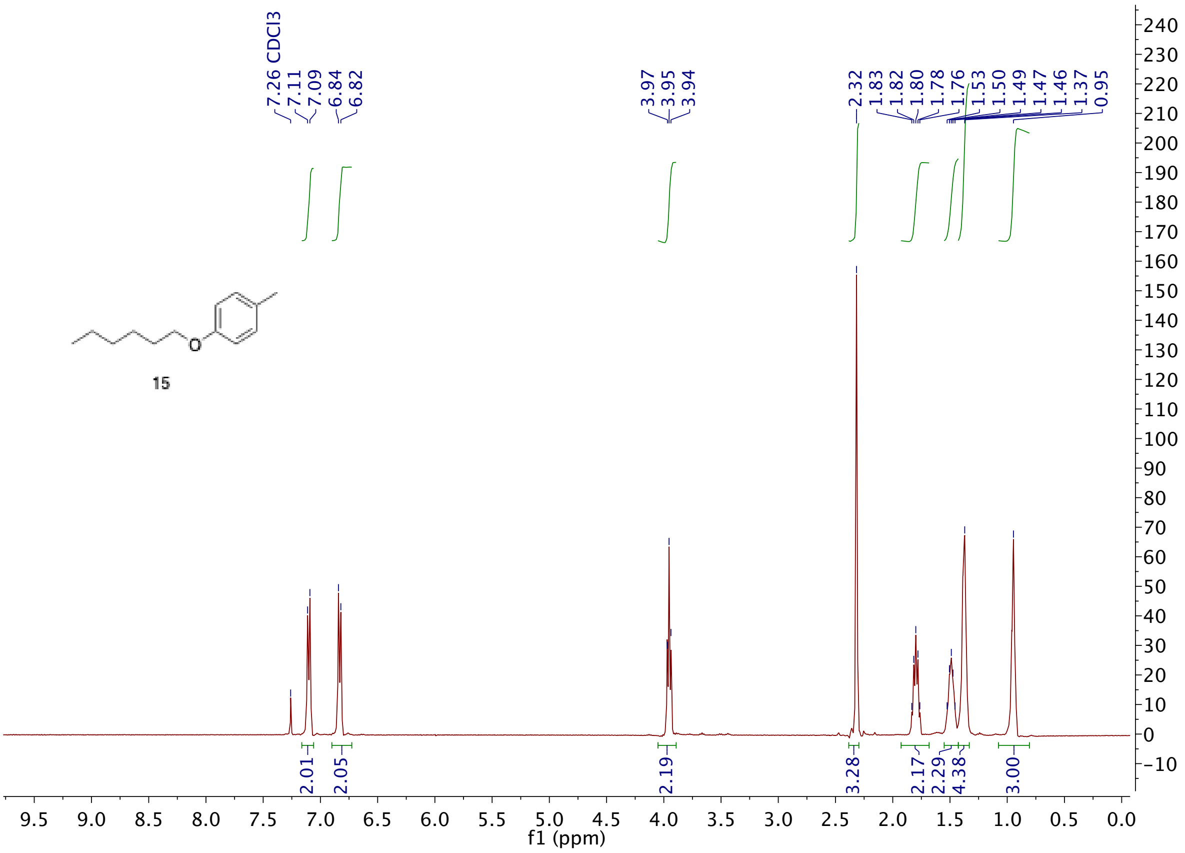

7.26 CDCl3
7.11
7.09
6.84
6.82
3.97
3.95
3.94
2.32
1.83
1.82
1.80
1.78
1.76
1.53
1.50
1.49
1.47
1.46
1.37
0.95
15
2.01
2.05
2.19
3.28
2.17
2.29
4.38
3.00
f1 (ppm)


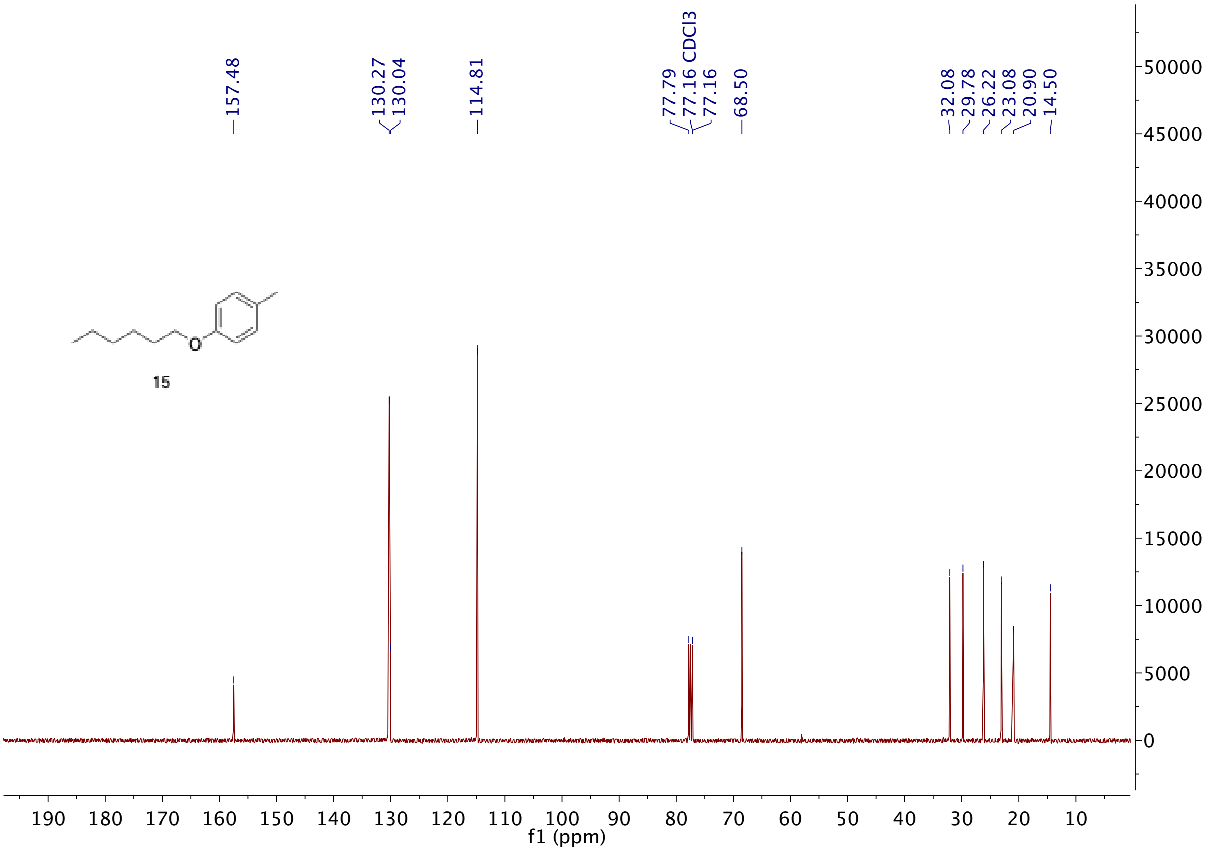

157.48
130.27
130.04
114.81
77.79
77.16 CDCl3
77.16
68.50
32.08
29.78
26.22
23.08
20.90
14.50
15
f1 (ppm)

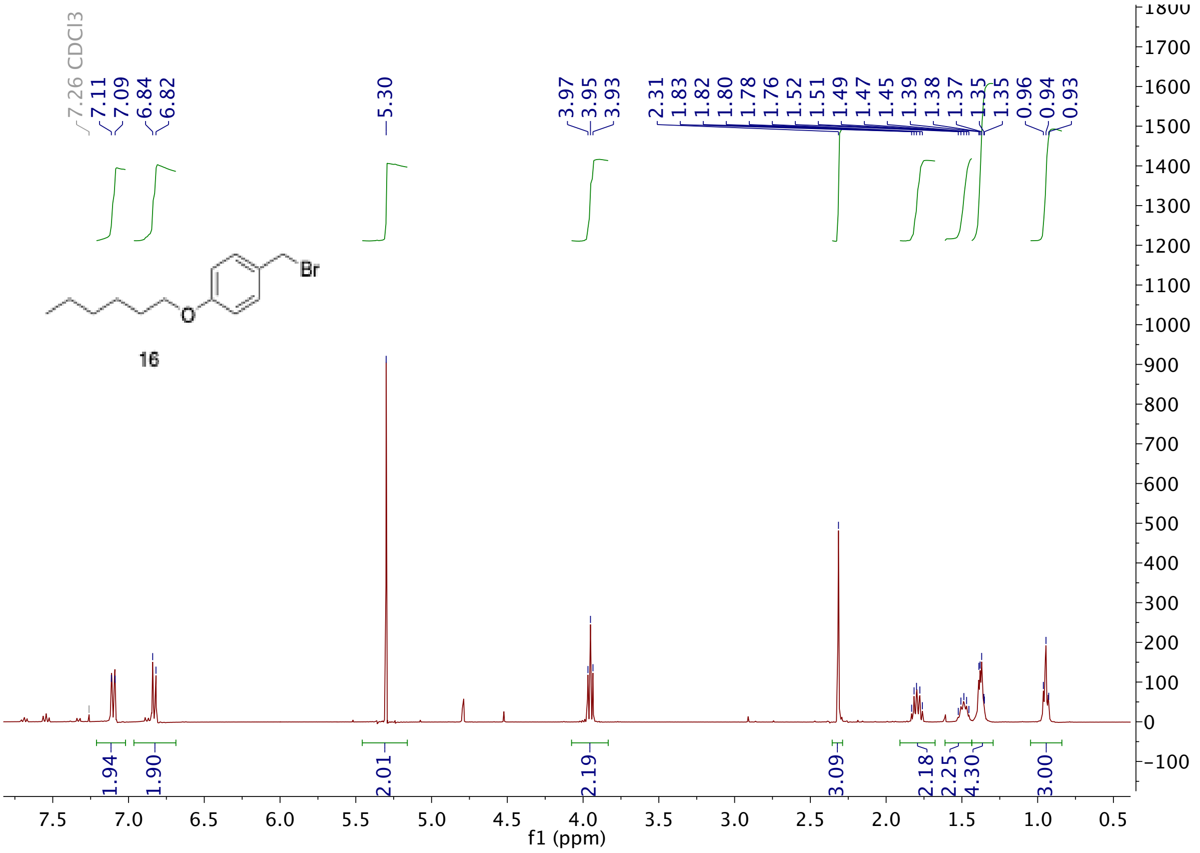
7.26 CDCl3
7.11
7.09
6.84
6.82
5.30
3.97
3.95
3.93
2.31
1.83
1.82
1.80
1.78
1.76
1.52
1.51
1.49
1.47
1.45
1.39
1.38
1.37
1.35
1.35
0.96
0.94
0.93
Br
O
16
1.94
1.90
2.01
2.19
3.09
2.18
2.25
4.30
3.00
f1 (ppm)

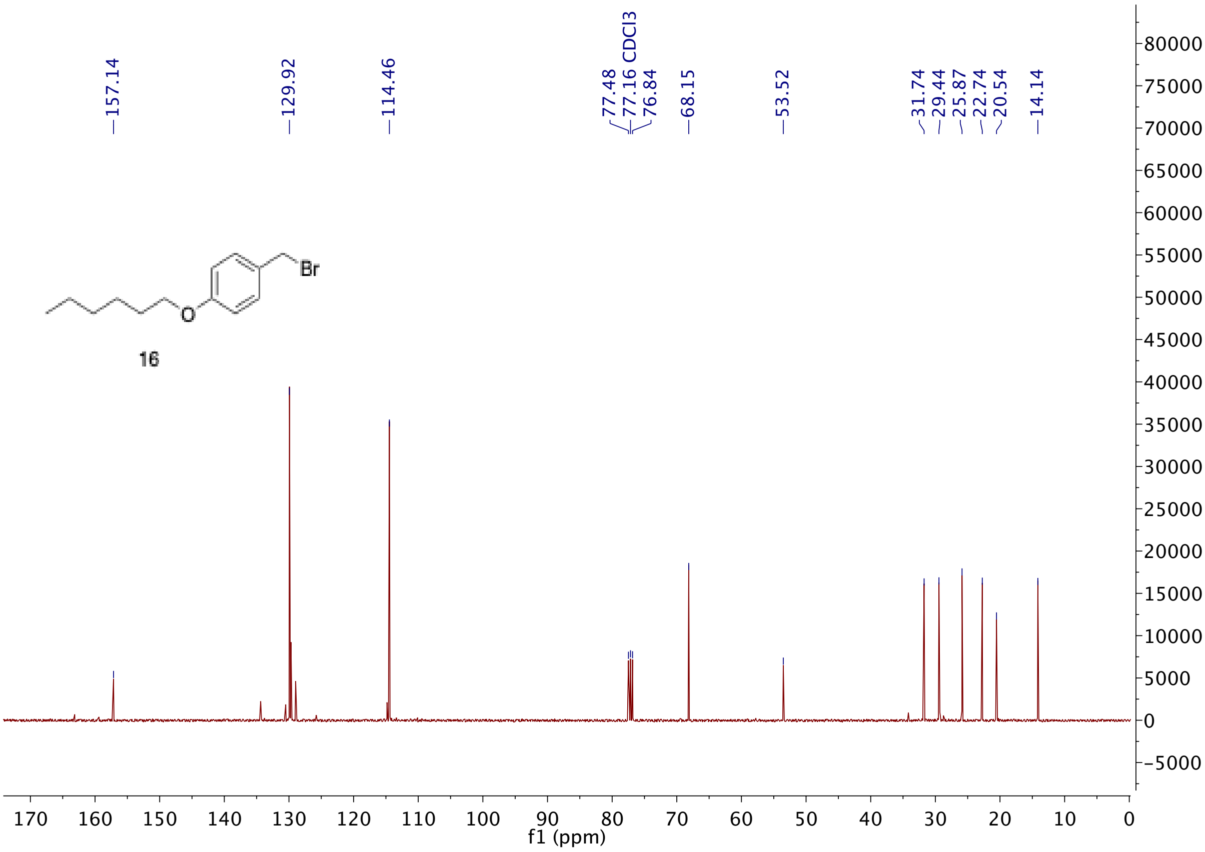
157.14
129.92
114.46
77.48
77.16 CDCl3
76.84
68.15
53.52
31.74
29.44
25.87
22.74
20.54
14.14
Br
O
16
f1 (ppm)

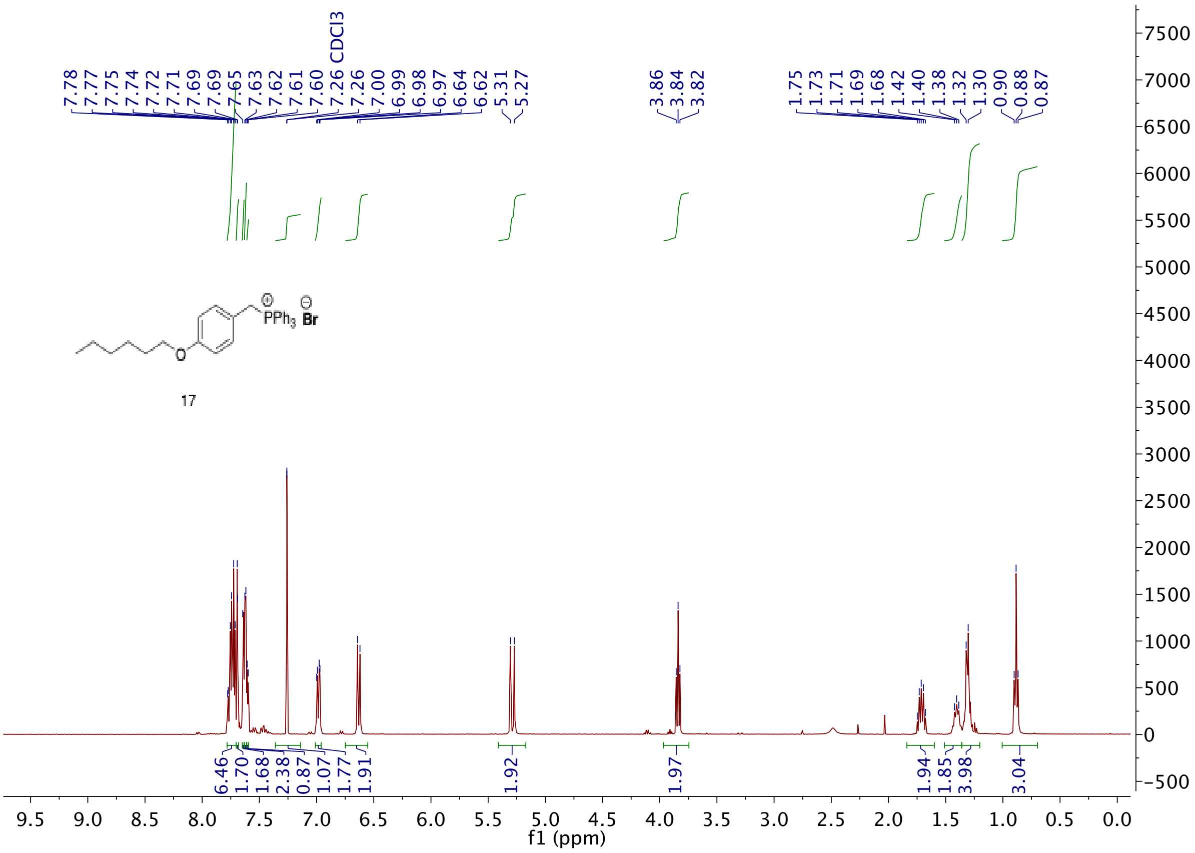
7.78 7.77 7.75 7.74 7.72 7.71 7.69 7.69 7.65 7.63 7.62 7.61 7.60 7.26 CDCl3 7.26 7.00 6.99 6.98 6.97 6.64 6.62 5.31 5.27
3.86 3.84 3.82
1.75 1.73 1.71 1.69 1.68 1.42 1.40 1.38 1.32 1.30 0.90 0.88 0.87
PPh3 Br
O
17
6.46 1.70 1.68 2.38 0.87 1.07 1.77 1.91 1.92 1.97 1.94 1.85 3.98 3.04
f1 (ppm)

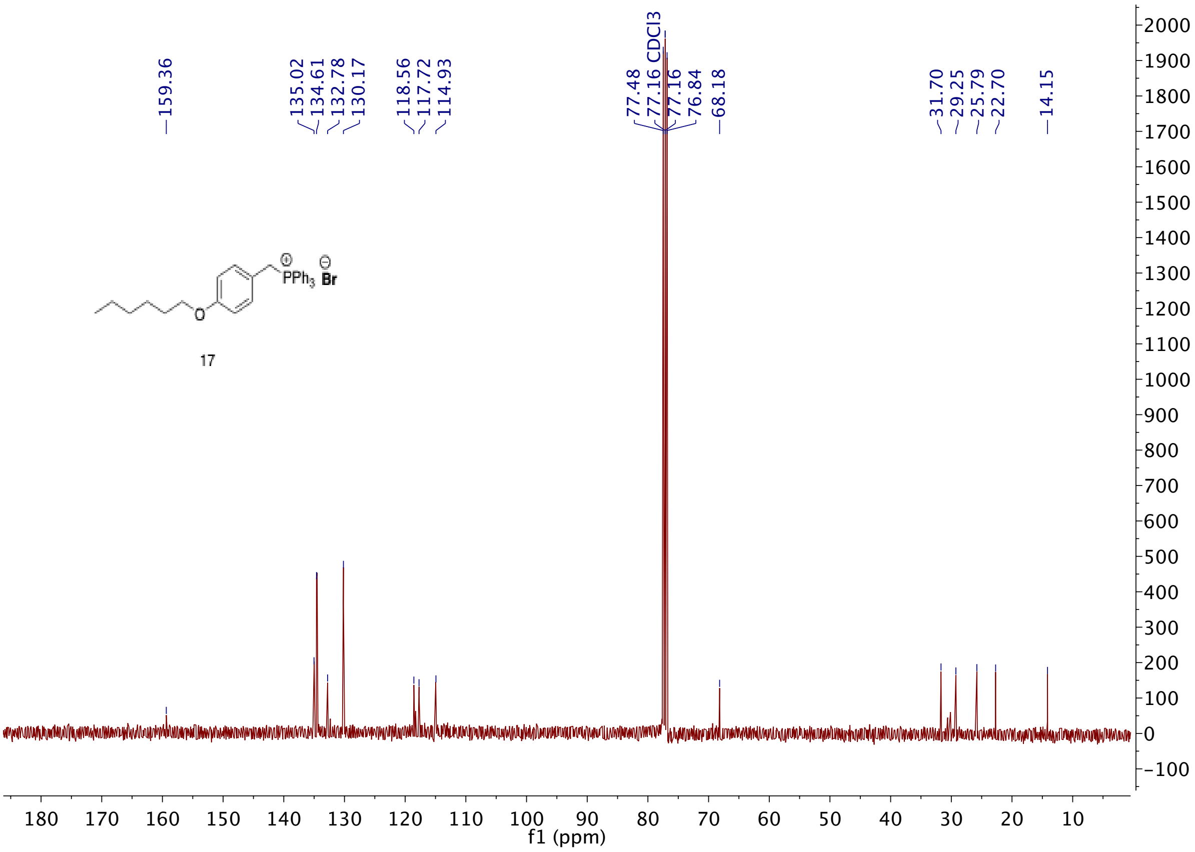
159.36
135.02 134.61 132.78 130.17
118.56 117.72 114.93
77.48 77.16 CDCl3 77.16 76.84 68.18
31.70 29.25 25.79 22.70 14.15
PPh3 Br
O
17
f1 (ppm)

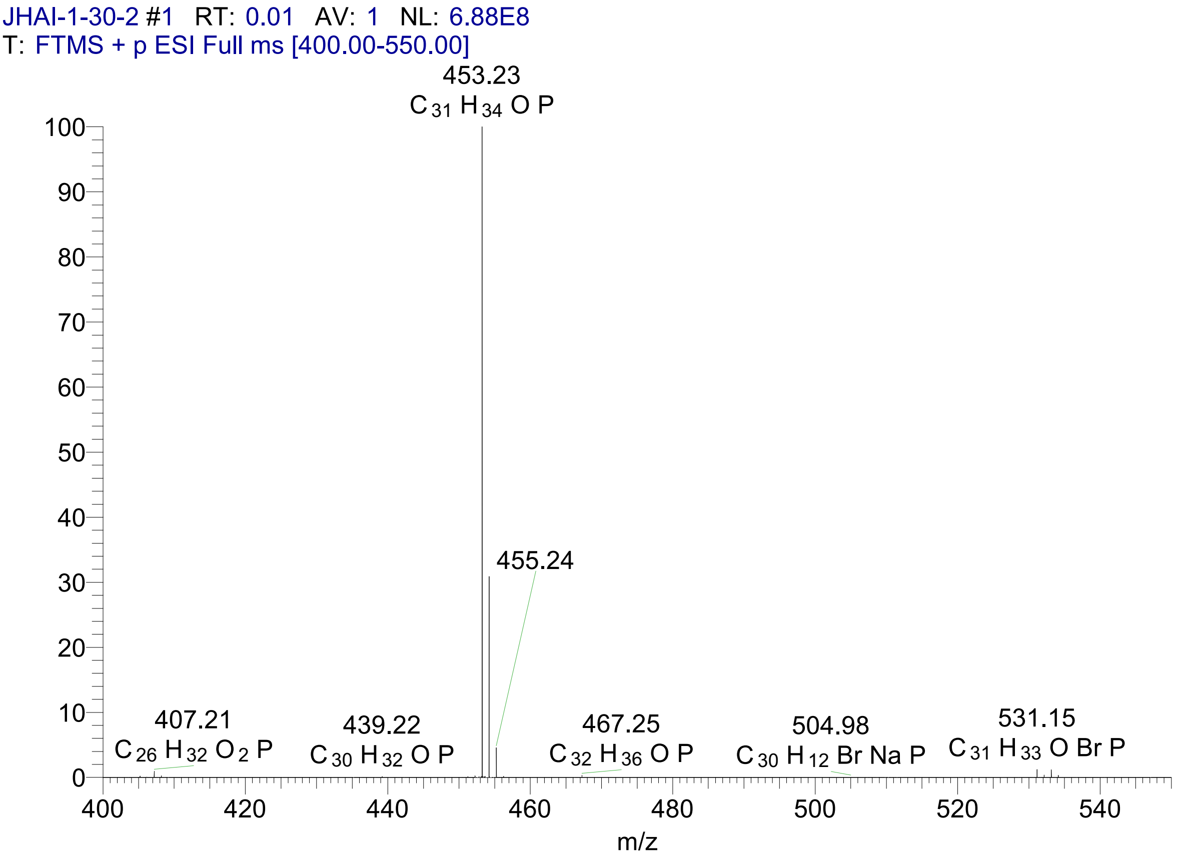
JHAI-1-30-2 #1 RT: 0.01 AV: 1 NL: 6.88E8
T: FTMS + p ESI Full ms [400.00-550.00]
453.23
C 31 H 34 O P
455.24
407.21
C 26 H 32 O 2 P
439.22
C 30 H 32 O P
467.25
C 32 H 36 O P
504.98
C 30 H 12 Br Na P
531.15
C 31 H 33 O Br P
m/z

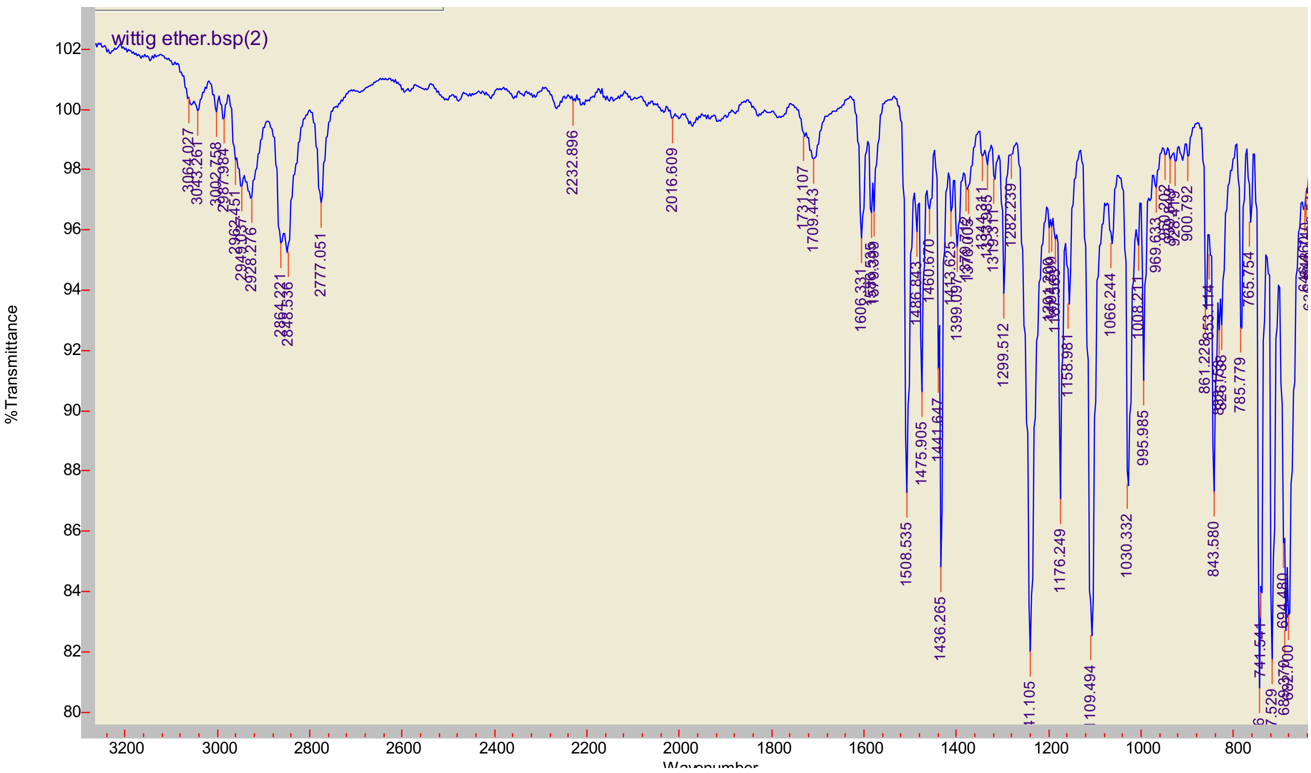
wittig ether.bsp(2)
%Transmittance
Wavenumber

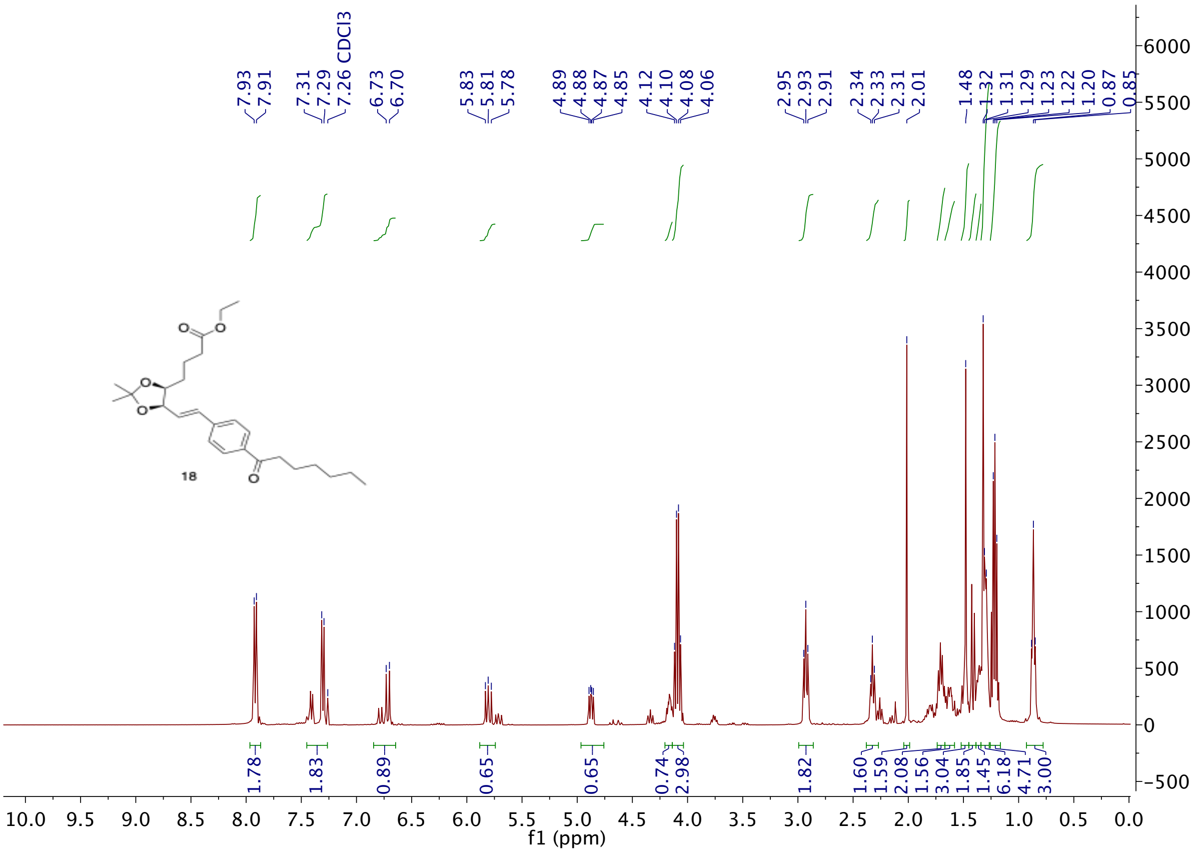

7.93
7.91
7.31
7.29
7.26 CDCl3
6.73
6.70
5.83
5.81
5.78
4.89
4.88
4.87
4.85
4.12
4.10
4.08
4.06
2.95
2.93
2.91
2.34
2.33
2.31
2.01
1.48
1.32
1.31
1.29
1.23
1.22
1.20
0.87
0.85
18
1.78
1.83
0.89
0.65
0.65
0.74
2.98
1.82
1.60
1.59
2.08
1.56
3.04
1.85
1.45
6.18
4.71
3.00
f1 (ppm)


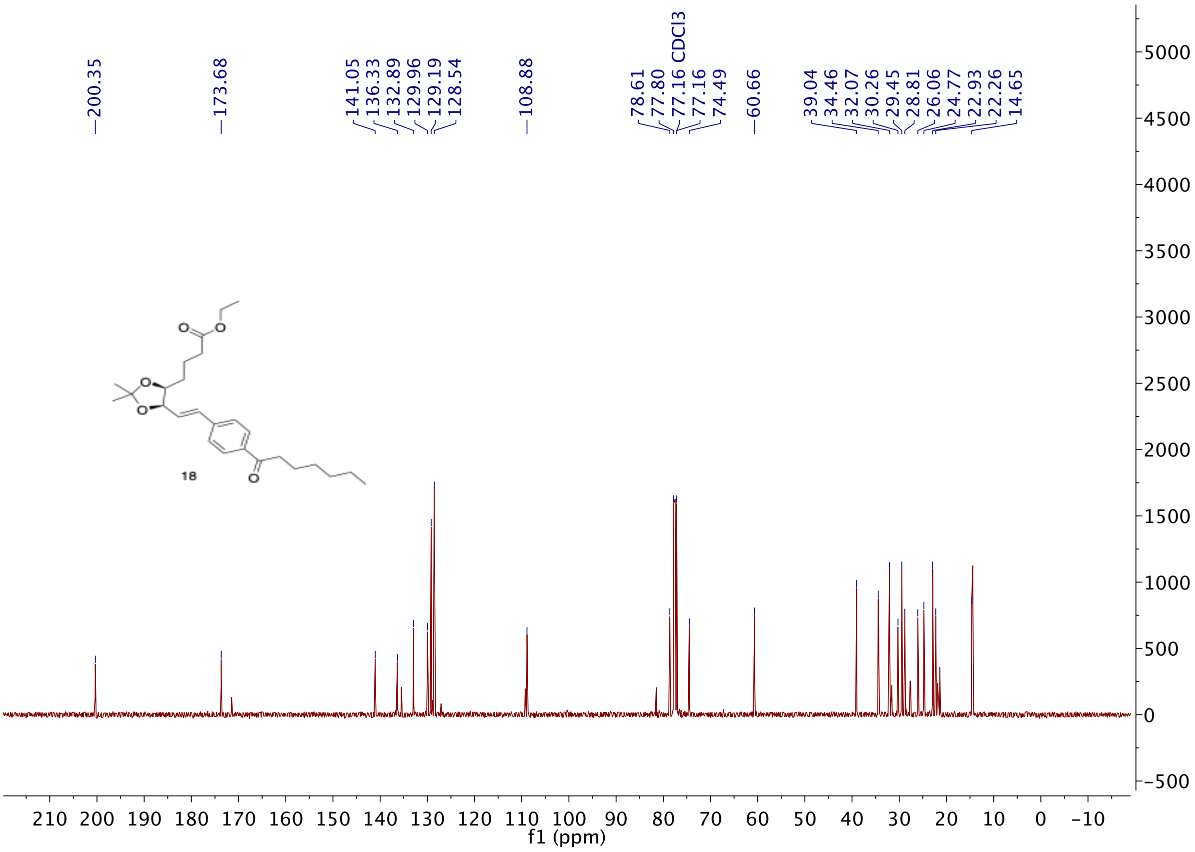

200.35
173.68
141.05
136.33
132.89
129.96
129.19
128.54
108.88
78.61
77.80
77.16 CDCl3
77.16
74.49
60.66
39.04
34.46
32.07
30.26
29.45
28.81
26.06
24.77
22.93
22.26
14.65
18
f1 (ppm)

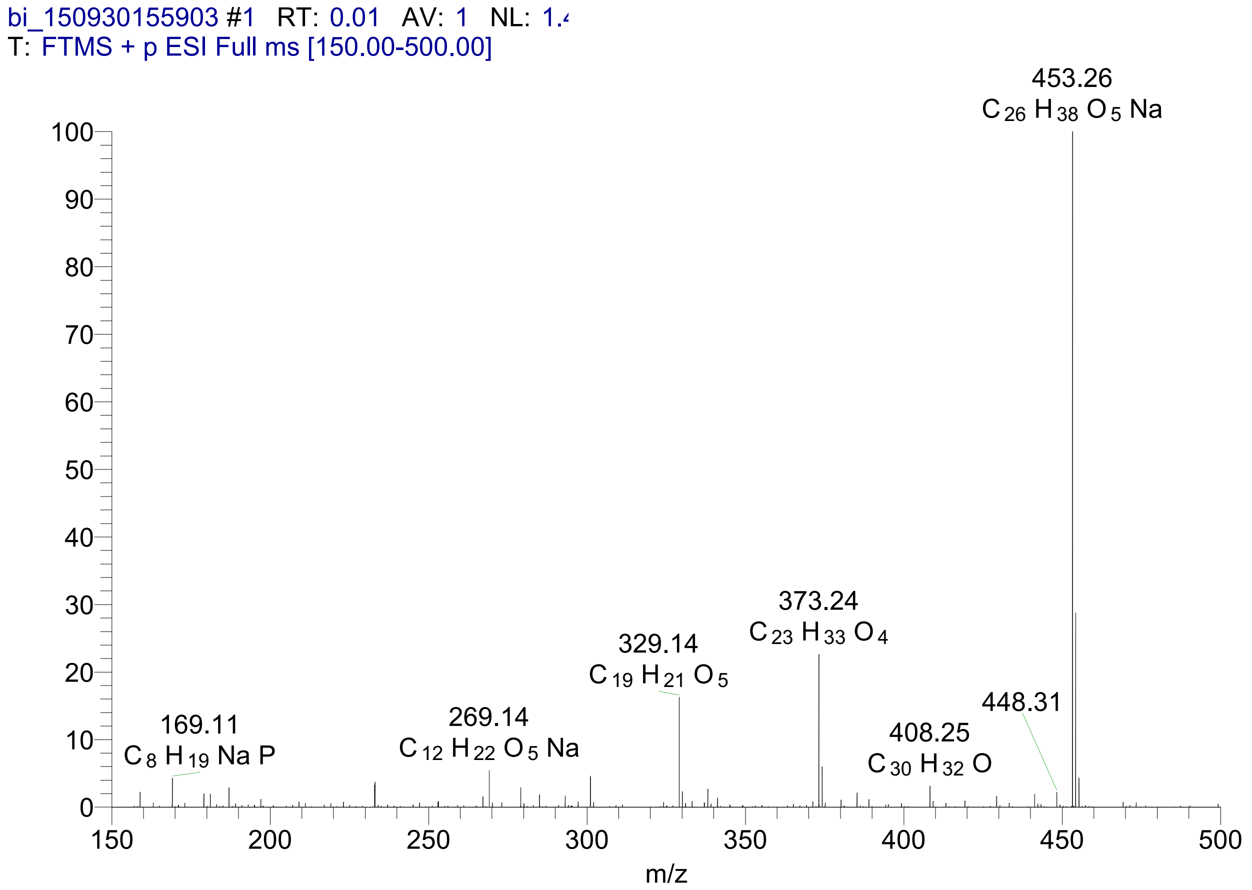

bi_150930155903 #1 RT: 0.01 AV: 1 NL: 1.4
T: FTMS + p ESI Full ms [150.00-500.00]
453.26
C 26 H 38 O 5 Na
373.24
C 23 H 33 O 4
329.14
C 19 H 21 O 5
269.14
C 12 H 22 O 5 Na
169.11
C 8 H 19 Na P
408.25
C 30 H 32 O
448.31
m/z


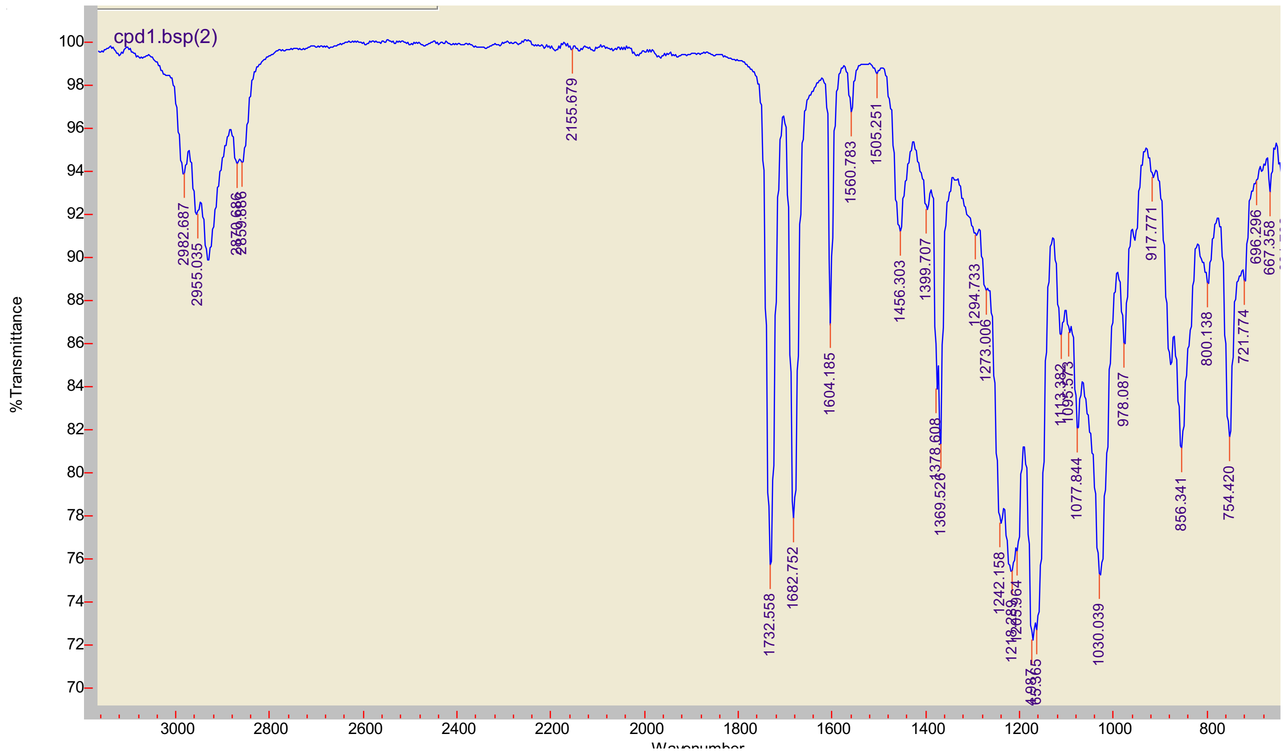

cpd1.bsp(2)
%Transmittance
2982.687
2955.035
2155.679
1732.558
1682.752
1604.185
1560.783
1505.251
1456.303
1399.707
1378.608
1369.526
1294.733
1273.006
1242.158
1030.039
1077.844
978.087
917.771
856.341
800.138
754.420
721.774
696.296
667.358

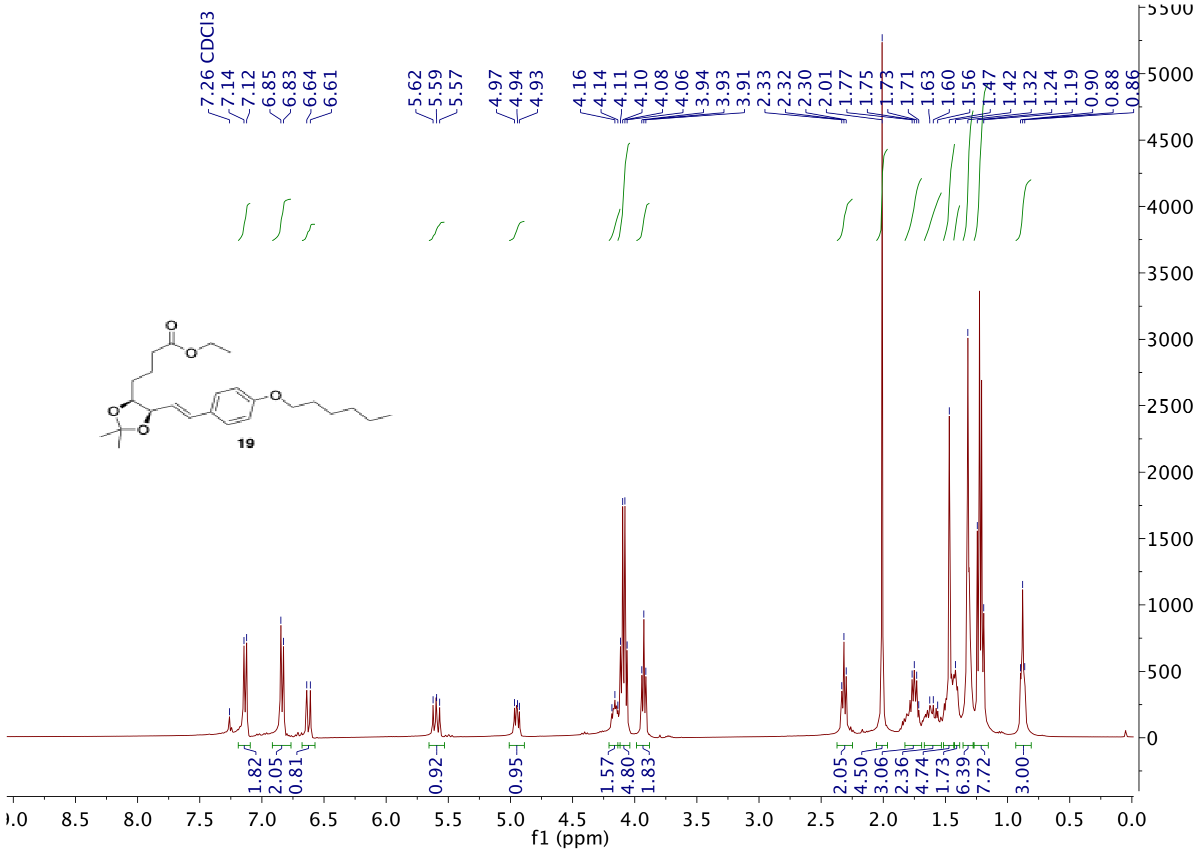

7.26 CDCl3
7.14
7.12
6.85
6.83
6.64
6.61
5.62
5.59
5.57
4.97
4.94
4.93
4.16
4.14
4.11
4.10
4.08
4.06
3.94
3.93
3.91
2.33
2.32
2.30
2.01
1.77
1.75
1.73
1.71
1.63
1.60
1.56
1.47
1.42
1.32
1.24
1.19
0.90
0.88
0.86
19
1.82
2.05
0.81
0.92
0.95
1.57
4.80
1.83
2.05
4.50
3.06
2.36
4.74
1.73
6.39
7.72
3.00
f1 (ppm)


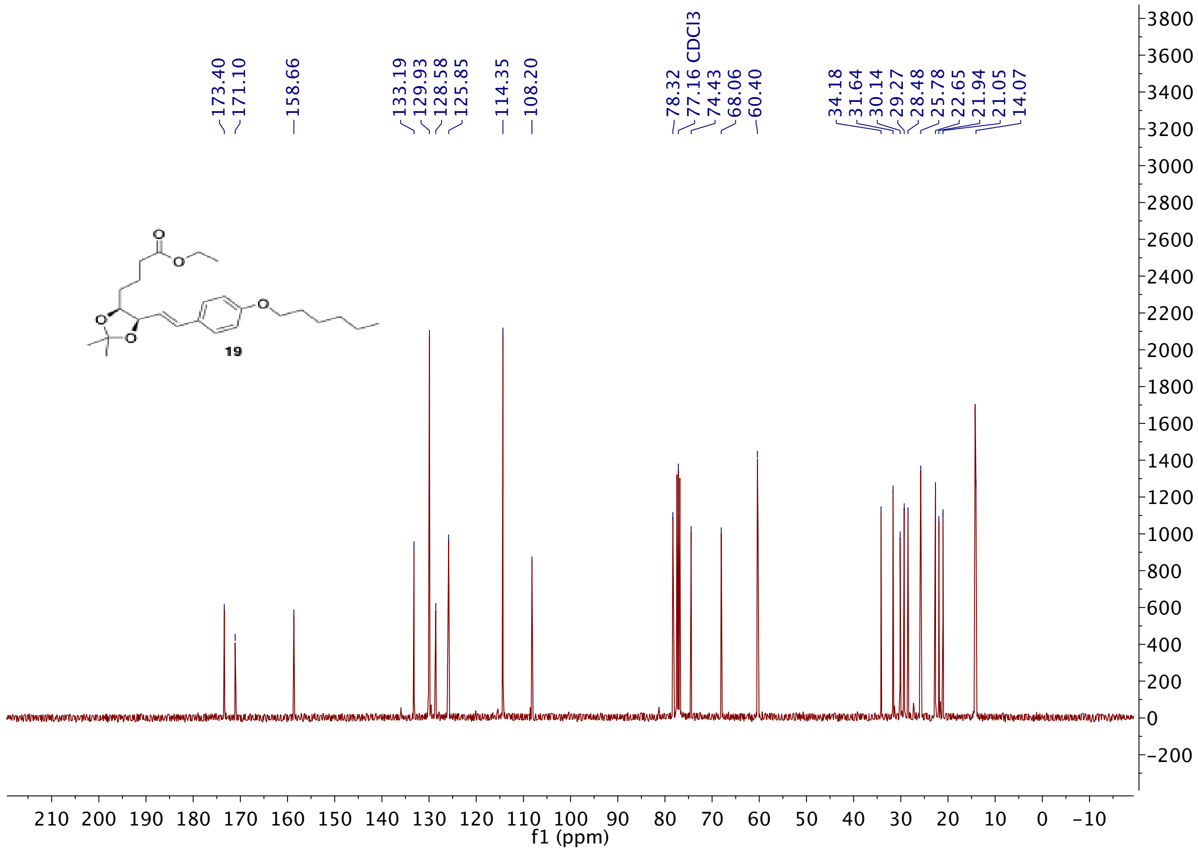

173.40
171.10
158.66
133.19
129.93
128.58
125.85
114.35
108.20
78.32
77.16 CDCl3
74.43
68.06
60.40
34.18
31.64
30.14
29.27
28.48
25.78
22.65
21.94
21.05
14.07
19
f1 (ppm)

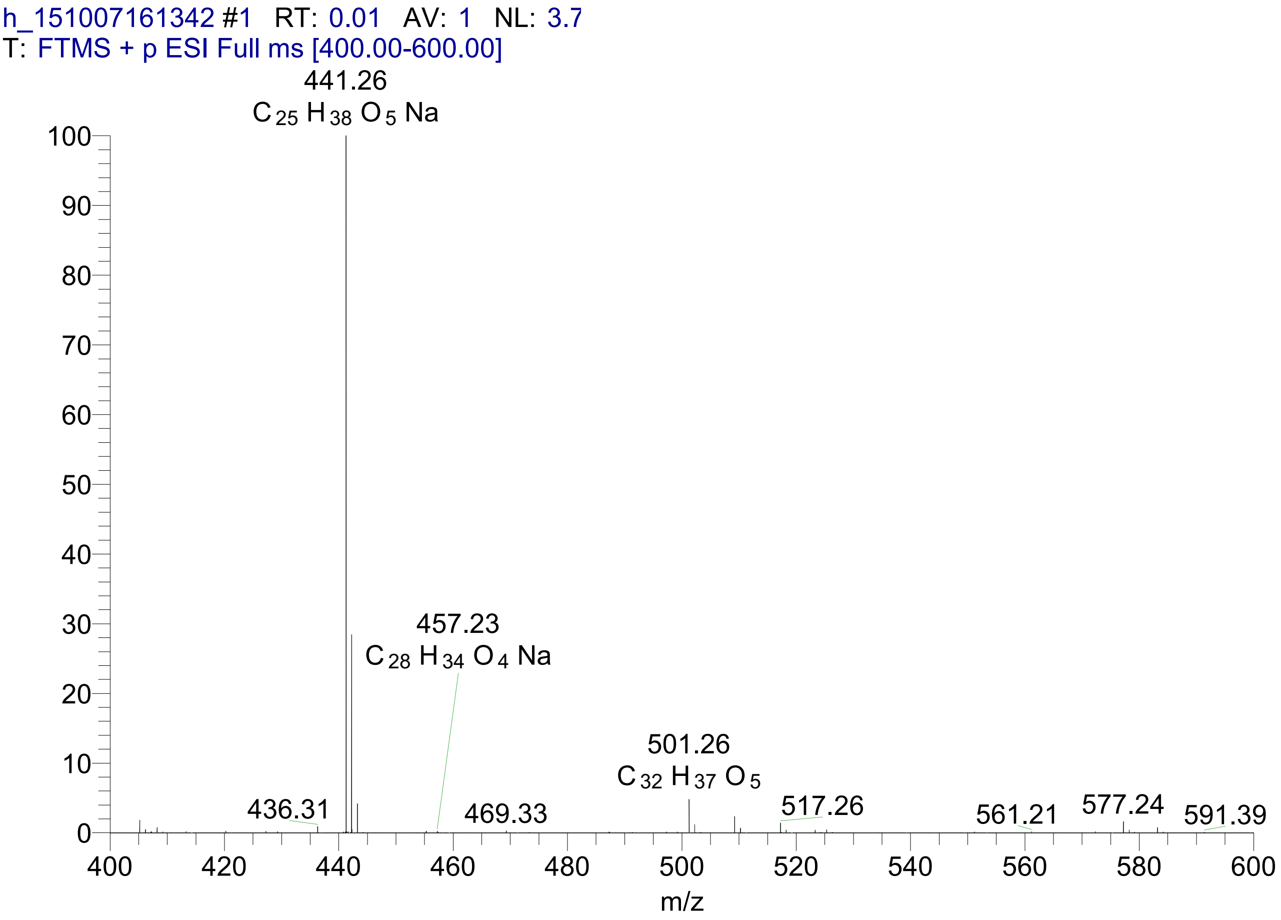
h_151007161342 #1 RT: 0.01 AV: 1 NL: 3.7
T: FTMS + p ESI Full ms [400.00-600.00]
441.26
C 25 H 38 O 5 Na
457.23
C 28 H 34 O 4 Na
501.26
C 32 H 37 O 5
436.31
469.33
517.26
561.21
577.24
591.39
m/z

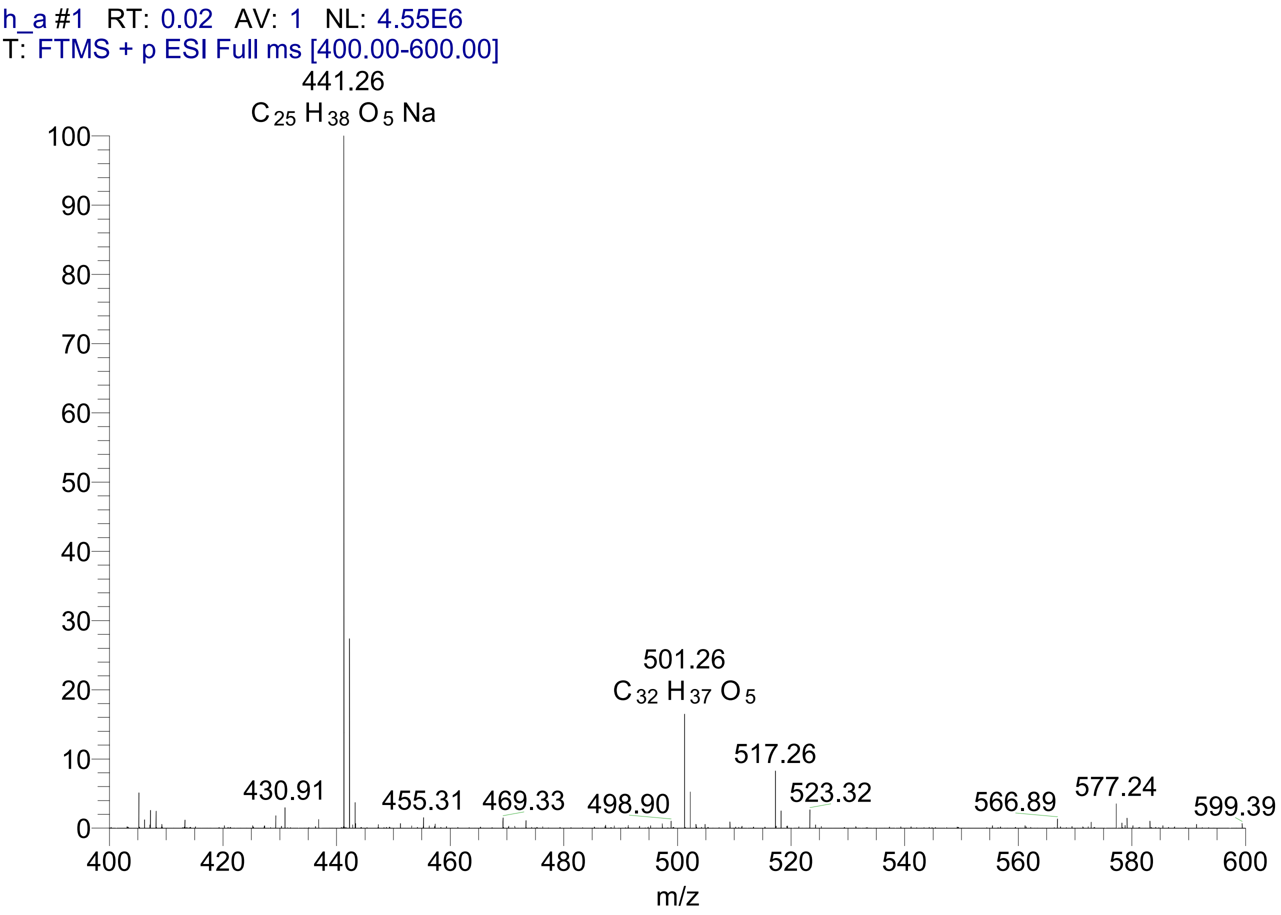
h_a #1 RT: 0.02 AV: 1 NL: 4.55E6
T: FTMS + p ESI Full ms [400.00-600.00]
441.26
C 25 H 38 O 5 Na
501.26
C 32 H 37 O 5
430.91
455.31
469.33
498.90
517.26
523.32
566.89
577.24
599.39
m/z

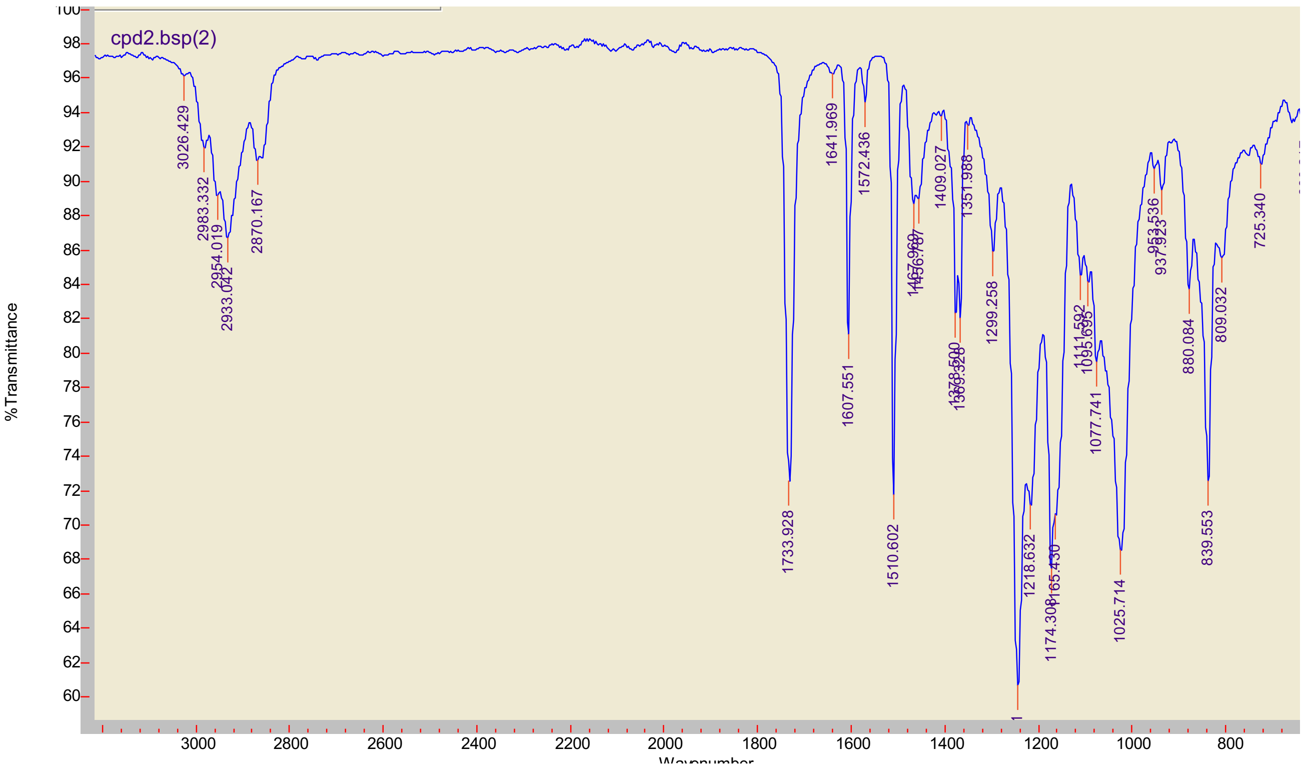
cpd2.bsp(2)
%Transmittance
Wavenumber
3026.429
2983.332
2954.019
2933.042
2870.167
1733.928
1641.969
1607.551
1572.436
1510.602
1409.027
1351.988
1299.258
1218.632
1174.308
1165.430
1077.741
1025.714
953.536
880.084
839.553
809.032
725.340

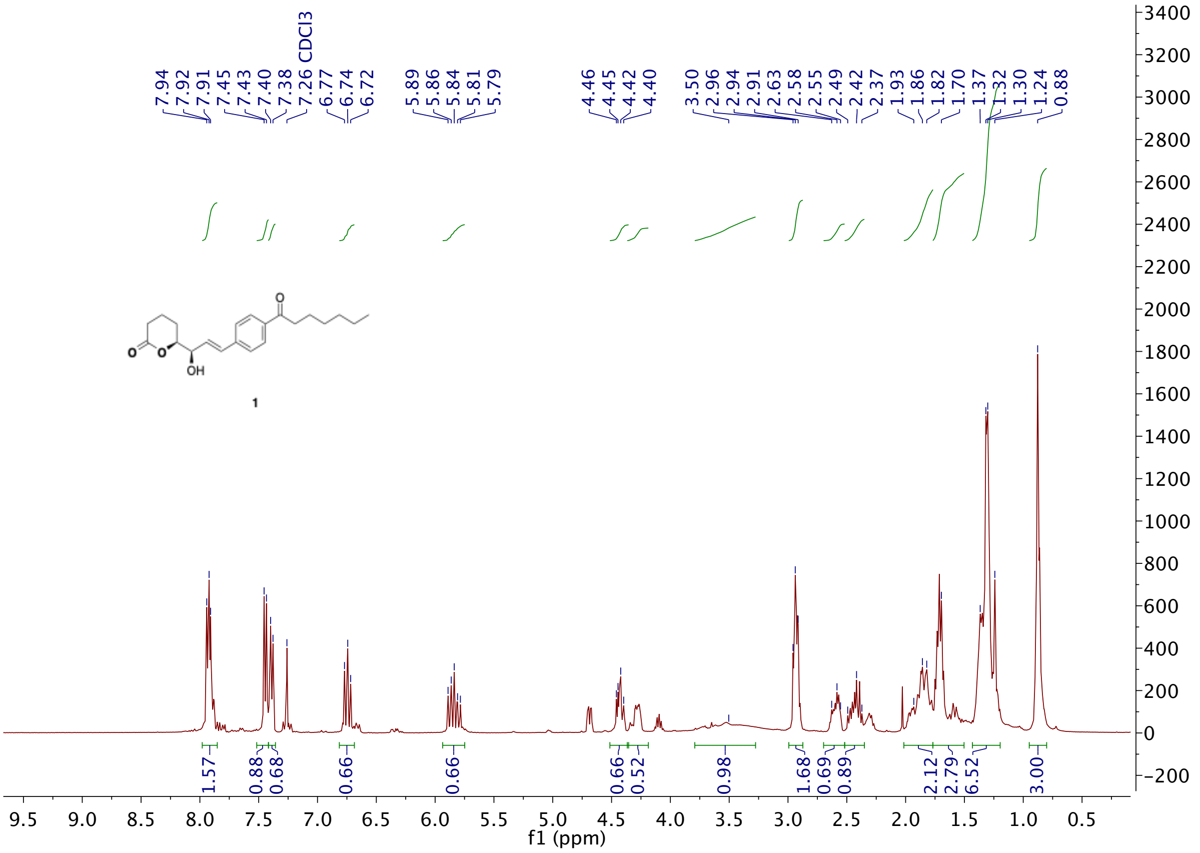
CDCl3
7.94
7.92
7.91
7.45
7.43
7.40
7.38
7.26
6.77
6.74
6.72
5.89
5.86
5.84
5.81
5.79
4.46
4.45
4.42
4.40
3.50
2.96
2.94
2.91
2.63
2.58
2.55
2.49
2.42
2.37
1.93
1.86
1.82
1.70
1.37
1.32
1.30
1.24
0.88
OH
1
1.57
0.88
0.68
0.66
0.66
0.66
0.52
0.98
1.68
0.69
0.89
2.12
2.79
6.52
3.00
f1 (ppm)

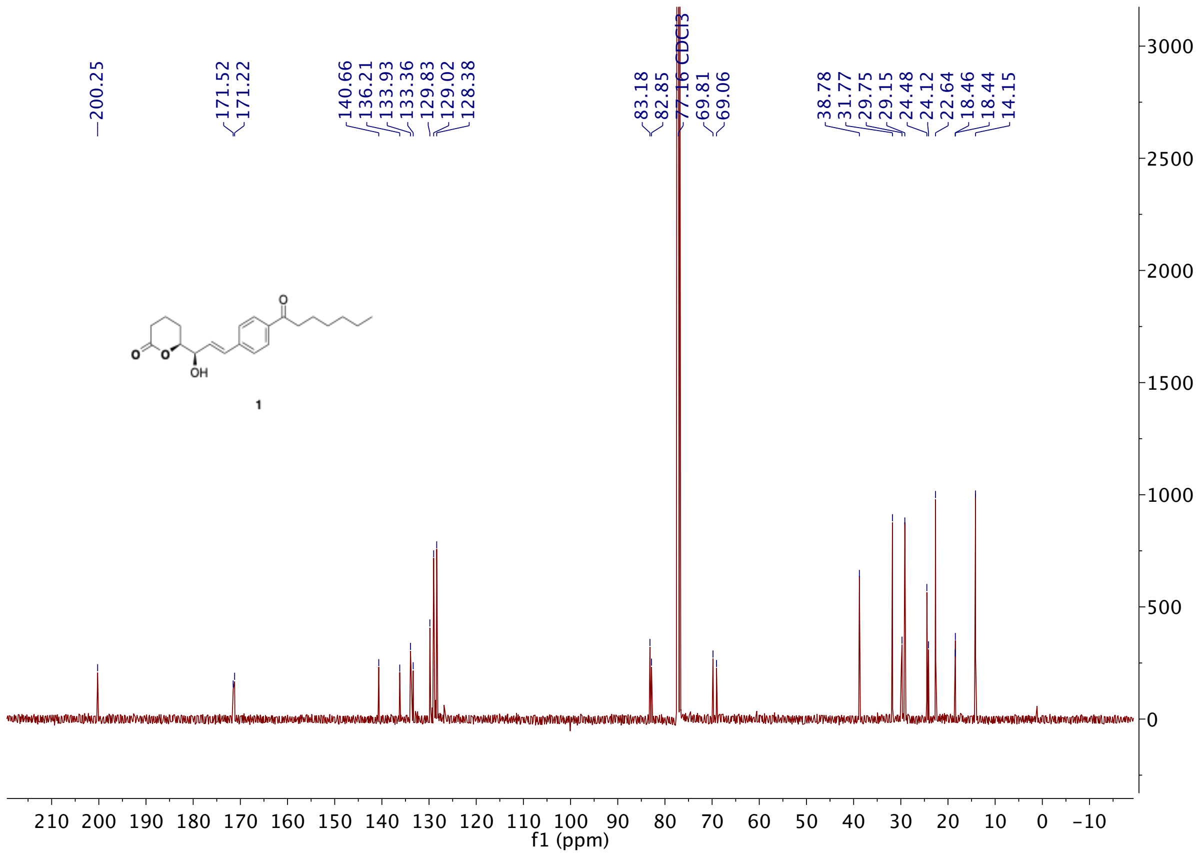

200.25
171.52
171.22
140.66
136.21
133.93
133.36
129.83
129.02
128.38
83.18
82.85
77.16 CDCl3
69.81
69.06
38.78
31.77
29.75
29.15
24.48
24.12
22.64
18.46
18.44
14.15
1
f1 (ppm)


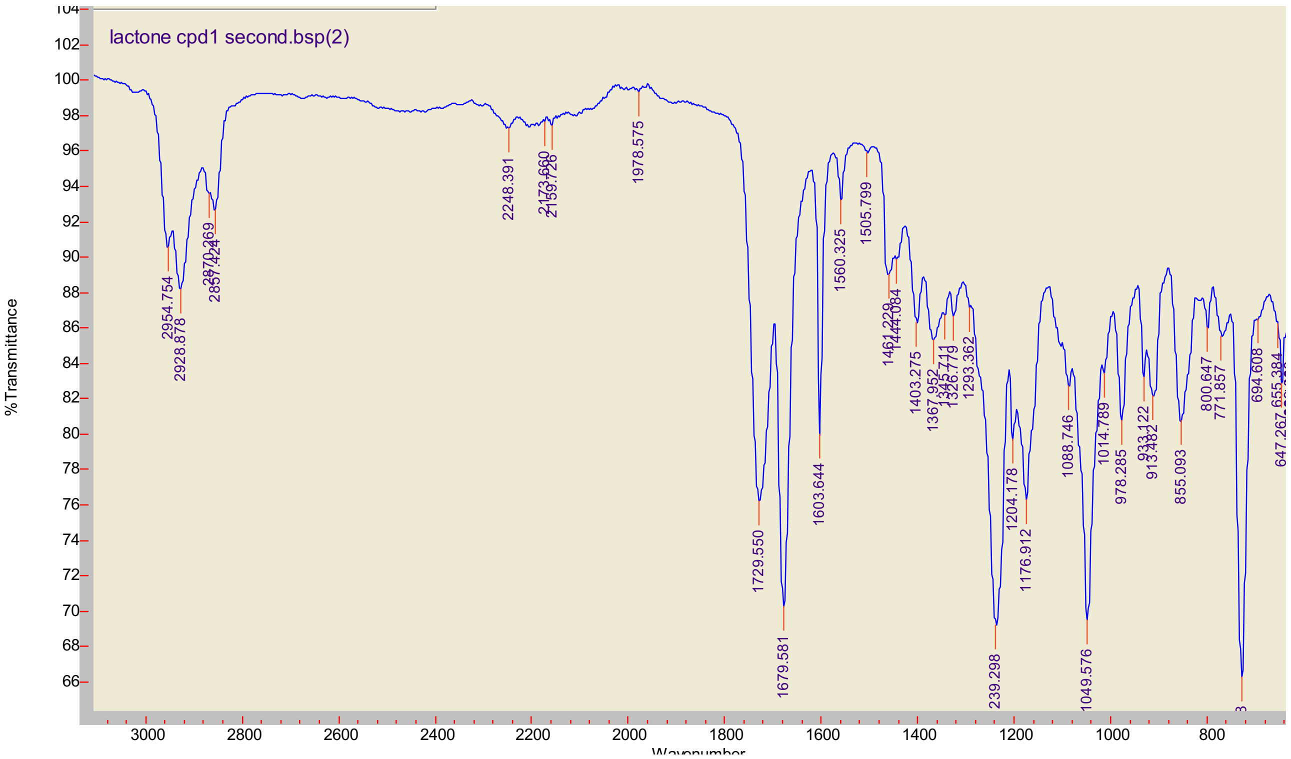

lactone cpd1 second.bsp(2)
%Transmittance
Wavenumber

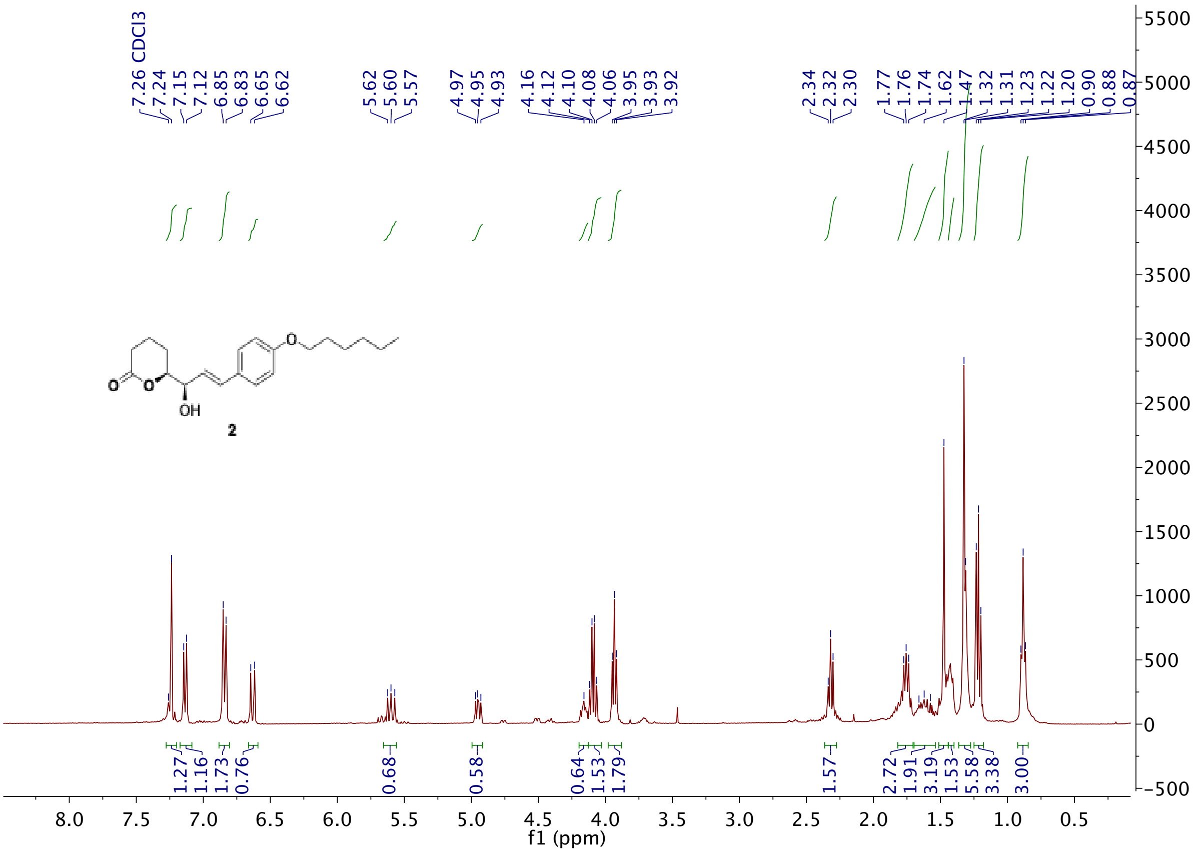
7.26 CDCl3
7.24
7.15
7.12
6.85
6.83
6.65
6.62
5.62
5.60
5.57
4.97
4.95
4.93
4.16
4.12
4.10
4.08
4.06
3.95
3.93
3.92
2.34
2.32
2.30
1.77
1.76
1.74
1.62
1.47
1.32
1.31
1.23
1.22
1.20
0.90
0.88
0.87
OH
O
O
O
2
1.27
1.16
1.73
0.76
0.68
0.58
0.64
1.53
1.79
1.57
2.72
1.91
3.19
1.53
5.58
3.38
3.00
f1 (ppm)

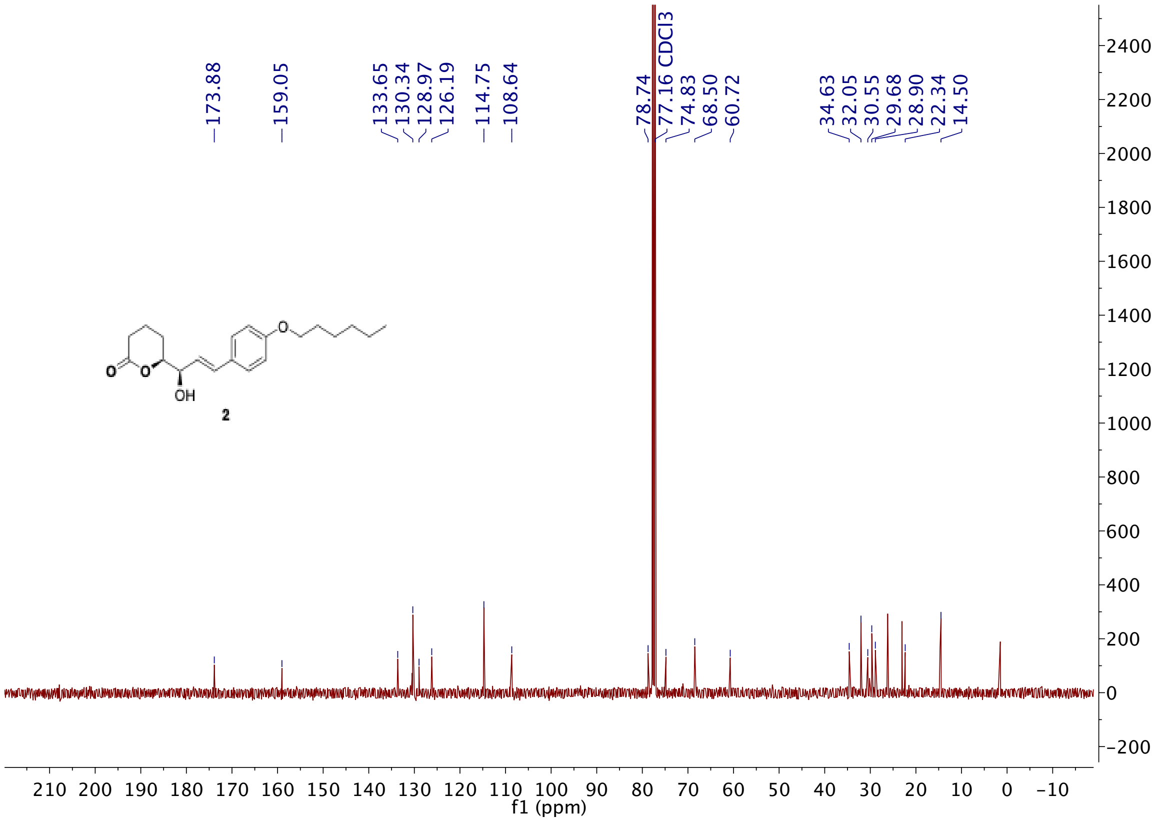
173.88
159.05
133.65
130.34
128.97
126.19
114.75
108.64
78.74
77.16 CDCl3
74.83
68.50
60.72
34.63
32.05
30.55
29.68
28.90
22.34
14.50
OH
O
O
O
2
f1 (ppm)

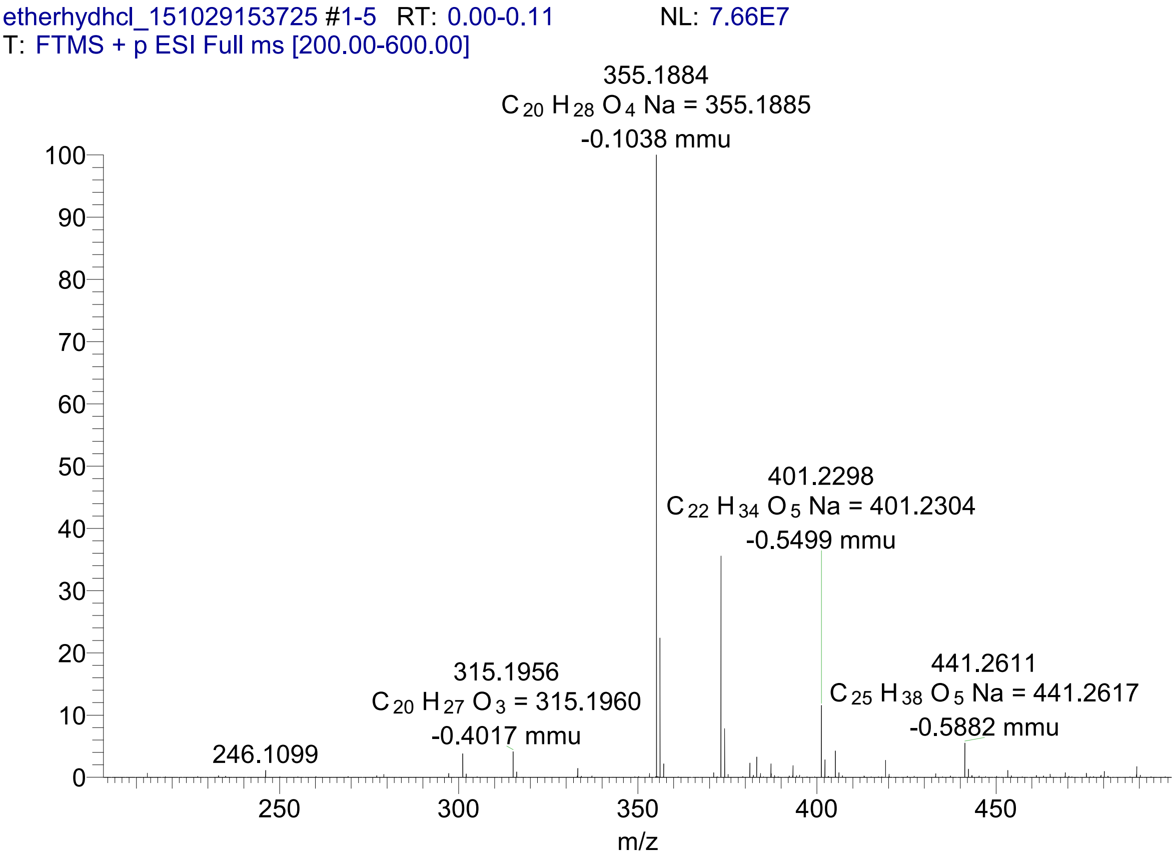
etherhydhcl_151029153725 #1-5 RT: 0.00-0.11 NL: 7.66E7
T: FTMS + p ESI Full ms [200.00-600.00]
355.1884
C 20 H 28 O 4 Na = 355.1885
-0.1038 mmu
401.2298
C 22 H 34 O 5 Na = 401.2304
-0.5499 mmu
315.1956
C 20 H 27 O 3 = 315.1960
-0.4017 mmu
441.2611
C 25 H 38 O 5 Na = 441.2617
-0.5882 mmu
246.1099
m/z

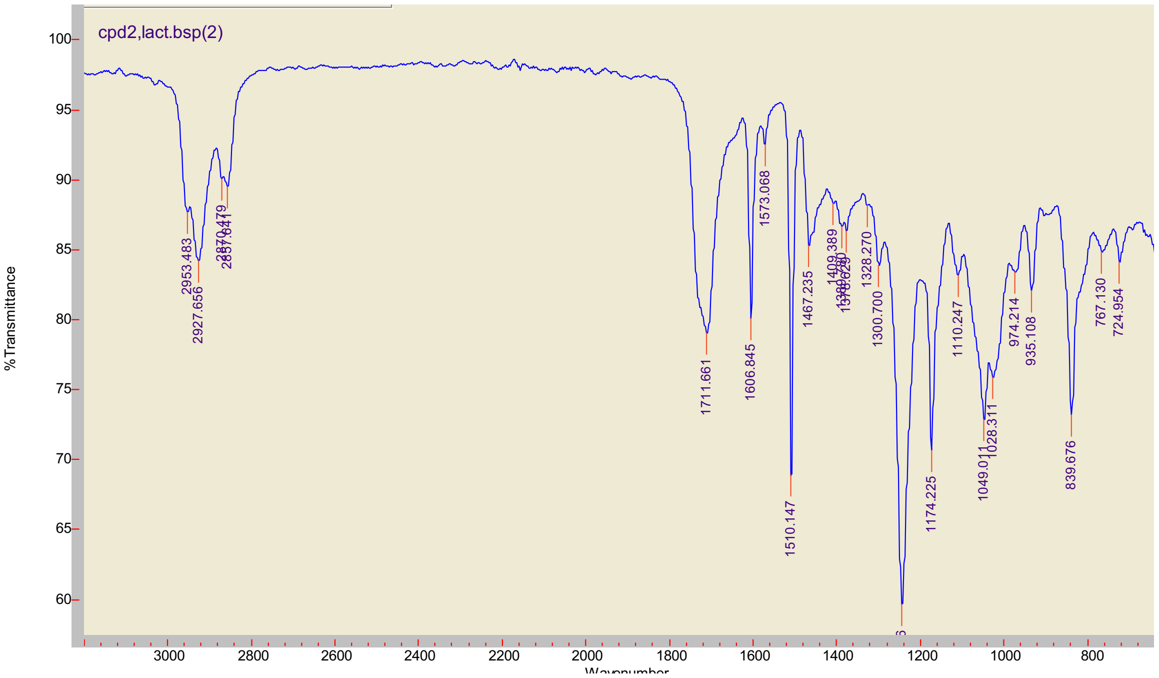
cpd2,lact.bsp(2)
%Transmittance
2953.483
2927.656
1711.661
1606.845
1573.068
1510.147
1467.235
1409.389
1328.270
1300.700
1174.225
1110.247
1049.011
1028.311
974.214
935.108
839.676
767.130
724.954

## References:


1. Silva M, Correia-Neves M. Neutrophils and macrophages: the main partners of phagocyte cell systems. *Front Immunol*. 2012; doi: 10.3389/fimmu.2012.00174
2. Duffy CD, Guiry PJ. Recent advances in the chemistry and biology of stable synthetic Lipoxin analogues. *MedChemComm.* 2010; 1: 249-65.
3. van Lent PL, Licht R, Dijkman H, Holthuysen AE, Berden JH, van den Berg WB. Uptake of apoptotic leukocytes by synovial lining macrophages inhibits immune complex-mediated arthritis. *J Leukoc Biol.* 2001; 70: 708-14.
4. Vandivier RW, Fadok VA, Ogden CA, Hoffmann PR, Brain JD, Accurso FJ, Fisher JH, Greene KE, Henson PM. Impaired clearance of apoptotic cells from cystic fibrosis airways. *Chest.* 2002; 121: 89s
5. Gaipl US, Kuhn A, Sheriff A, Munoz LE, Franz S, Voll RE, Kalden JR, Herrmann M. Clearance of apoptotic cells in human SLE. *Curr Dir Autoimmun.* 2006; 9: 173-87.
6. O'Sullivan TP, Vallin KS, Shah ST, Fakhry J, Maderna P, Scannell M., Sampaio AL, Perretti M, Godson C, Guiry PJ. Aromatic lipoxin A4 and lipoxin B4 analogues display potent biological activities. *J Med Chem.* 2007; 50: 5894-902.
7. Serhan CN, Hamberg M, Samuelsson B. Lipoxins: novel series of biologically active compounds formed from arachidonic acid in human leukocytes. *Proc Natl Acad Sci USA.* 1984; 81: 5335-39.
8. McMahon B, Godson C. Lipoxins: endogenous regulators of inflammation. *Am J Physiol Renal Physiol.* 2004; 286: F189-F201.
9. Brink C, Dahlén S-E, Drazen J, Evans JF, Hay DWP, Nicosia S, Serhan CN, Shimizu T, Yokomizo T. International Union of Pharmacology XXXVII. Nomenclature for Leukotriene and Lipoxin Receptors. *Pharmacol Rev.* 2003; 55: 195.
10. Ye RD, Boulay F, Wang JM, Dahlgren C, Gerard C, Parmentier M, Serhan CN, Murphy PM. International Union of Basic and Clinical Pharmacology. LXXIII. Nomenclature for the Formyl Peptide Receptor (FPR) Family. *Pharmacol Rev.* 2009; 61: 119.
11. Chiang N., Serhan CN, Dahlén S-E, Drazen JM, Hay DWP, Rovati GE, Shimizu T, Yokomizo T, Brink C. The Lipoxin receptor ALX: Potent ligand-specific and stereoselective actions in vivo. *Pharmacol Rev.* 2006; 58: 463.
12. Petasis NA, Akritopoulou-Zanze I, Fokin VV, Bernasconi G, Keledjian R, Yang R, Uddin J, Nagulapalli KC, Serhan CN. Design, synthesis and bioactions of novel stable mimetics of lipoxins and aspirin-triggered lipoxins. *Prostaglandins Leukot Essent Fatty Acids.* 2005; 73: 301-21.
13. Petasis N, Keledjian R, Sun Y-P, Nagulapalli KC, Tjonahen E, Yang R, Serhan CN. Design and synthesis of benzo-lipoxin A4 analogs with enhanced stability and potent anti-inflammatory properties. *Bioorg Med Chem Lett.* 2008; 18: 1382-7.
14. Phillips ED, Chang H-F, Holmquist CR, McCauley JP. Synthesis of methyl (5S,6R,7E,9E,11Z,13E,15S)-16-(4-fluorophenoxy)-5,6,15-trihydroxy-7,9,11,13-hexadecatetraenoate, an analogue of 15R-lipoxin A4. *Bioorg Med Chem Lett.* 2003; 13: 3223-6.
15. Yadav JS, Barma DK, Dutta D. Stereoselective Total Synthesis of 5(S), 6(R), 15(S)-Trihydroxy-7(E), 9(E), 11(Z),13(E)-Eicosatetraenoic Acid (Lipoxin A). *Tetrahedron Lett.* 1998; 39: 143-6.
16. Rodríguez AR, Spur BW. Total synthesis of aspirin-triggered 15-epi-lipoxin A4. *Tetrahedron Lett.* 2001; 42: 6057-60.
17. Nicolaou KC, Marron BE., Veale CA, Webber S, Serhan CN. Total synthesis of novel geometric isomers of lipoxin A4 and lipoxin B4. *J Org Chem.* 1989; 54: 5527-35.
18. Nicolao KC, Veale CA, Webber SE, Katerinopoulos, H. Stereocontrolled total synthesis of lipoxins A. *J Am Chem Soc.*1985; 107: 7515-8.

19. Duffy CD, Maderna P, McCarthy C, Loscher CE, Godson C, Guiry PJ. Synthesis and biological evaluation of pyridine-containing Lipoxin A4 analogues. *ChemMedChem.* 2010; 5: 517-22.
20. O'Sullivan TP, Vallin KSA, Ali Shah ST, Fakhry J, Maderna P, Scannell M, Sampaio ALF, Perretti M, Godson C, Guiry PJ. Aromatic Lipoxin A4 and Lipoxin B4 analogues display potent biological activities. *J Med Chem.* 2007; 50: 5894-902.
21. Haberlin GG, McCarth C., Doran R, Loscher CE, Guiry PJ. Asymmetric synthesis and biological evaluation of 1,3- and 1,4-disubstituted benzo-type lipoxin A4 analogues. *Tetrahedron.* 2014; 70: 6859-69.
22. Hasegawa A, Kiso M. Acetonation of 2-(acylamino)-2-deoxy-d-glucoses. *Carbohydr. Res.* 1978; 63: 91-8.
23. Wolfrom ML, Diwadkar AB, Gelas J, Horton D. A new method of acetonation. Synthesis of 4,6-O-isopropylidene-D-glucopyranose. *Carbohydr. Res.* 1974; 35: 87-96.
24. Takeo K, Murata Y, Kitamura S. A facile synthesis of 4-O-allyl-d-xylopyranose and its use in the preparation of xylo-oligosaccharides. *Carbohydr. Res.* 1992; 224: 311-8.
25. Pneumonia. In: World Health Organization. http://www.who.int/mediacentre/factsheets/fs331/en/. Accessed March 2017.
26. Liu R, Zhang P, Gan T, Cook JM. Regiospecific Bromination of 3-Methylindoles with NBS and Its Application to the Concise Synthesis of Optically Active Unusual Tryptophans Present in Marine Cyclic Peptides. *J Org Chem.* 1997; 62: 7447-56.
27. Aitken, R. A., Jethwa, S. J., Richardson, N. V., and Slawin, A. M. Z. (2016) Regioselective bromination of 1,4-dimethoxy-2,3-dimethylbenzene and conversion into sulfur-functionalised benzoquinones. *Tetrahedron Lett.* 2016; 57: 1563-6.
28. Lin H-C, Jiang M-D, Wu S-C, et al. Self-assembly of H-bonded side-chain and cross-linking copolymers containing diblock-copolymeric donors and single/double H-bonded light-emitting acceptors. *J Polym Sci Part A Polym Chem*. 2009; 47:4685-702.
29. Clish CB, O'Brien JA, Gronert K, Stahl GL, Petasis NA, Serhan CN. Local and systemic delivery of a stable aspirin-triggered lipoxin prevents neutrophil recruitment in vivo. *Proc Natl Acad Sci U S A*. 1999; 96: 8247-52.
30. Tseng M-C, Kan H-C, Chu Y-H. Reactivity of trihexyl(tetradecyl)phosphonium chloride, a room-temperature phosphonium ionic liquid. *Tetrahedron Lett*. 2007; 48: 9085-9.
31. Podgoršek A, Stavber S, Zupan M, Iskra J. Visible light induced 'on water' benzylic bromination with N-bromosuccinimide. *Tetrahedron Lett*. 2006; 47: 1097-9.
32. Miljanić S, Frkanec L, Meić Z, Žinić M. Photoinduced gelation by stilbene oxalyl amide compounds. *Langmuir*. 2005; 21: 2754-60.
